# Functional and muscle recovery after critical illness: current and future nutritional, physical and metabolic strategies

**DOI:** 10.1016/j.aicoj.2026.100137

**Published:** 2026-08-24

**Authors:** Alexandre Pierre, Steve Lancel, Josepha Bertrac, Claire Bourel, Thierry Van Der Linden, Raphael Favory, Sebastien Preau

**Affiliations:** a Univ. Lille, Inserm, CHU Lille, JUNIA, U1352-BioPrev-Bien Vieillir: de l'Inflammaging à la Prévention, Lille, France; b Department of Intensive Care, Hospital Group of the Catholic Institute of Lille, Saint Philibert Hospital, Catholic University of Lille, Lille, France; c Division of Intensive Care, Hôpital Roger Salengro, CHU de Lille, Lille, France; d Department of Intensive Care, Hospital Group of the Catholic Institute of Lille, FMMS-ETHICS EA7446, Saint Philibert Hospital, Catholic University of Lille, Lille, France

**Keywords:** ICU-acquired weakness, Post-intensive care syndrome, Critical illness, Sarcopenia, Anabolic resistance, Feeding responsiveness, Exercise responsiveness, Early mobilization, Nutritional support

## Abstract

Despite gains in survival, recovery from the physical consequences of critical illness has not improved. ICU-acquired weakness (ICUAW) develops in roughly half of mechanically ventilated patients and causes durable muscle dysfunction, reduced exercise capacity and curtailed autonomy that persist for years, with impact on quality of life and healthcare utilization.

The diagnostic framework remains limited: the Medical Research Council sum score is informative at ICU discharge but loses sensitivity during convalescence, and capturing the full deficit requires multidimensional assessment of muscle function, physical function and quality of life, in line with the PRACTICE core outcome set and the conceptual continuum from acute ICUAW to post-ICU sarcopenia. Two pillars structure current therapy — nutritional support and physical rehabilitation. Nutritional support: avoidance of severe hyperglycaemia is established, but large RCTs consistently show that escalating acute-phase calorie or protein delivery does not improve, and may worsen, outcomes, particularly in vulnerable subgroups such as those with acute kidney injury. This paradox is explained by the concept of feeding responsiveness: anabolic resistance blunts muscle protein synthesis in a way that cannot be overcome by dose escalation; as inflammation and metabolism recover, a catabolic-to-anabolic transition restores nutrient utilization, and dynamic biomarkers (urea trajectory, phosphate kinetics, insulin resistance index) may identify the catabolic-to-anabolic transition at the bedside. These insights translate into a four-phase individualized strategy spanning permissive underfeeding with conditional escalation, standard targets, protocol-guided individualized support during early (in-hospital) convalescence, and enriched nutrition with structured dietitian-led follow-up after discharge. Physical rehabilitation: early mobilization improves short-term outcomes — muscle strength at ICU discharge, duration of mechanical ventilation and length of stay — but recent trials, notably TEAM, show that augmented-dose mobilization does not translate into long-term functional benefit and may harm identifiable subgroups, mirroring the nutritional paradox. The concept of exercise responsiveness — captured by clinical signatures of non-response (diabetes, low achievable ICU Mobility Scale, illness severity) and molecular determinants (persistent inflammation, mitochondrial and proteostatic dysfunction) — supports a four-phase physiology-informed prescription, from safety-first early mobilization to home-based adapted physical activity sustaining a continuous training stimulus. Beyond these two pillars, emerging metabolic support offers complementary, biology-targeted interventions still under preclinical and translational development. These include alternative oxidative substrates, autophagy and mitochondrial modulators, and anabolic agents — forcing anabolism acutely may be harmful, as growth hormone showed, but androgens, oestrogens and amino acid metabolites may merit testing in convalescence — alongside senolytics and cell-based approaches targeting impaired muscle regeneration.

The convergent results of dose-based interventions can be operationalized through the concepts of feeding and exercise responsiveness, which point to biology-guided individualization. Composite ready-to-feed and ready-to-train indicators integrating bedside biomarkers, muscle microbiopsy readouts and biological signatures, validated through adaptive platform trials, represent the next frontier toward synchronized restoration of muscle and functional recovery after critical illness.

## Introduction

Survival from critical illness has improved substantially over the past two decades, but it has revealed a dominant long-term burden: a durable impairment of physical and functional recovery. ICU-acquired weakness (ICUAW) develops in roughly half of mechanically ventilated patients and translates into persistent muscle dysfunction, reduced exercise capacity and curtailed autonomy long beyond hospital discharge. These impairments reduce health-related quality of life, increase caregiver burden, and elevate healthcare utilization, reframing critical illness as a true inflection point in the patient's life trajectory. The underlying pathobiology is beginning to be characterized, but clinical operationalization remains hampered by the absence of a consensual definition of post-ICU muscle weakness. The MRC sum score is informative at ICU discharge but loses sensitivity during convalescence: most survivors regain an MRC > 48 within weeks, yet retain measurable deficits in dynamometric strength, aerobic capacity and self-reported physical function for years. A multidimensional assessment integrating muscle function, physical function and quality of life is therefore required, making explicit the continuum from acute ICUAW to post-ICU sarcopenia.

Defining the patient and the relevant outcomes is only the first step. No pharmacological strategy has yet demonstrated a definitive clinical benefit for post-ICU muscle weakness, and the two operational pillars of recovery — nutritional support and physical rehabilitation — have so far not consistently improved long-term outcomes in randomized trials, even though physical rehabilitation yields demonstrable short-term gains. Unlike the muscle of a healthy individual recovering from acute injury, the critically ill and convalescent muscle harbours persistent molecular alterations that reshape its capacity to respond to feeding and exercise stimuli — a concept we will operationalize as feeding responsiveness and exercise responsiveness. Both constructs, and the derived ready-to-feed and ready-to-train indicators, are conceptual frameworks rather than validated clinical paradigms. Emerging metabolic support strategies — alternative oxidative substrates and autophagy modulators — are gaining attention as complementary, biology-targeted interventions, although clinical evidence remains preliminary.

The aim of this narrative review is twofold. First, we delineate the clinical magnitude and operational definition of the physical recovery deficit, with a multidimensional framework linking ICUAW to post-ICU sarcopenia. Second, we review current and emerging strategies for functional and muscle recovery — nutrition, physical rehabilitation and metabolic support — applying for each a consistent three-step structure: clinical evidence, underlying concept of responsiveness or biological rationale, and a phase-specific individualized strategy from ICU admission to home-based convalescence.

## Physical and functional recovery after critical illness

### ICU-acquired weakness and its impact on long-term outcomes

**Fig. 1:**
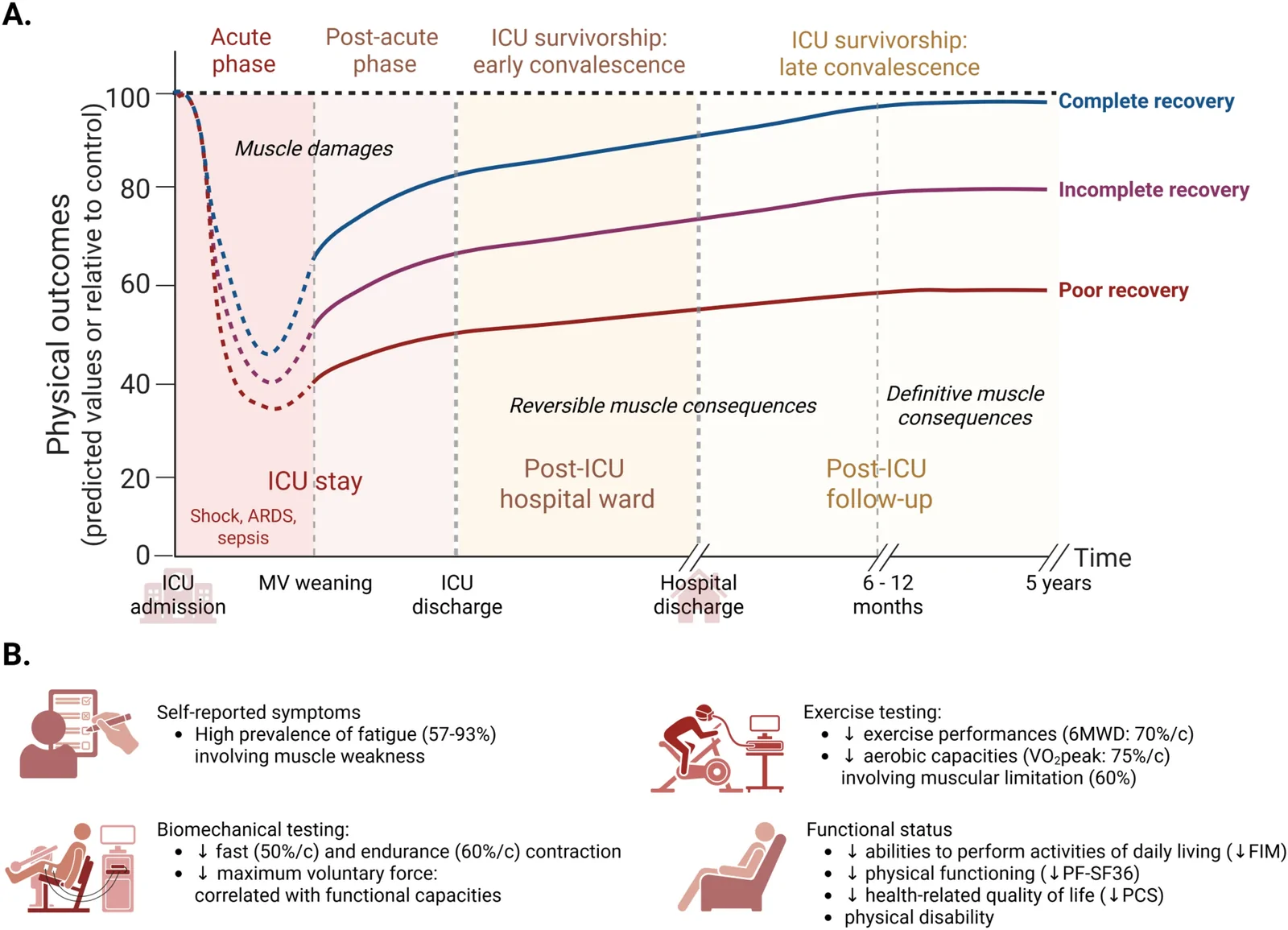
Trajectories and manifestations of long-term physical impairment in survivors after critical illness. A. Conceptual trajectory of physical outcomes after critical illness (shock, ARDS, sepsis): acute muscle injury during ICU stay, recovery through early and late convalescence, with heterogeneous trajectories from complete to poor recovery and a proportion of patients developing persistent muscle dysfunction. The trajectories labelled complete, incomplete and poor recovery are a conceptual schematisation of the heterogeneity observed in longitudinal cohorts; they illustrate the range of outcomes rather than define validated, threshold-based categories. B. Multidimensional consequences of post-ICU muscle dysfunction: patient-reported symptoms, biomechanical alterations, impaired exercise capacity, functional limitations, and health-related quality of life. %/c, percentage of control values (predicted or age/sex-matched healthy controls). ARDS, acute respiratory distress syndrome; MV, mechanical ventilation; 6MWD, 6-minute walk distance; VO₂peak, peak oxygen uptake; FIM, Functional Independence Measure; PF-SF36, physical functioning domain of the Short Form-36; PCS, physical component summary score.

ICUAW encompasses critical illness myopathy (CIM), critical illness polyneuropathy (CIP) and mixed forms (Table S1), which differ in pathophysiology and prognosis [1]. A predominant myopathic component appears to drive the sustained loss of force in survivors, whereas a significant polyneuropathic component is associated with slower and less complete neurological recovery [[2], [3], [4]]. The two may also respond differently to rehabilitation. ICUAW is tightly coupled to the physical component of post-intensive care syndrome (PICS) and to patient-centered outcomes after discharge [5]. Muscle weakness and functional limitations are common at hospital discharge and remain clinically meaningful during convalescence (Fig. 1). ICUAW not only worsens short-term outcomes (mechanical ventilation duration, length of stay, hospital mortality) [6,7] but also has lasting consequences, including long-term physical impairments and higher late mortality [[8], [9], [10]]. Physical function typically improves during the first 3–6 months and then plateaus for years [11]. One year after ICU discharge, maximal and endurance force are reduced by ∼50% and ∼40% versus healthy controls [2]. Fatigue affects 57–93% of survivors and is commonly associated with persistent muscle weakness [[12], [13], [14]], adversely affecting physical performance and quality of life [15]. Persistent physical and aerobic limitations have been reported up to five years: in the 5-year EPaNIC follow-up, six-minute walk distance reached ∼70% and peak oxygen uptake ∼75% of control values, with exercise limitations attributable to muscular factors in ∼60% of cases [[16], [17], [18]]. A substantial proportion of previously independent patients fail to regain pre-ICU functional status [19], and recovery trajectories remain heterogeneous [20]. Nearly two-thirds do not regain pre-ICU physical status at one year [17]; among sepsis survivors with functional dependence, only one-third recover independence [21]. Since the landmark ARDS cohort of Herridge et al. [22,23], long-term impairment of health-related quality of life has been well recognized, and Van Aerde et al. recently confirmed that persistent physical impairments strongly correlate with reduced health-related quality of life five years post-ICU [8]. At the health-system level, population-based analyses suggest that for many ARDS and septic shock survivors, critical illness represents an inflection point toward persistently elevated healthcare utilization.

Physical and functional recovery results from the interplay between pre-ICU health status, ICU complications, post-ICU consequences, and persistent molecular and cellular alterations of skeletal muscle, detailed below [1]. In EPaNIC survivors, altered muscle transcriptomic signatures persisted at five years and correlated with lower long-term physical capacities; these signatures were partly associated with potentially modifiable ICU exposures (glucocorticoids, early parenteral nutrition), supporting the relevance of metabolic and nutritional therapeutics [24,25].

### Physical health evaluation

**Fig. 2:**
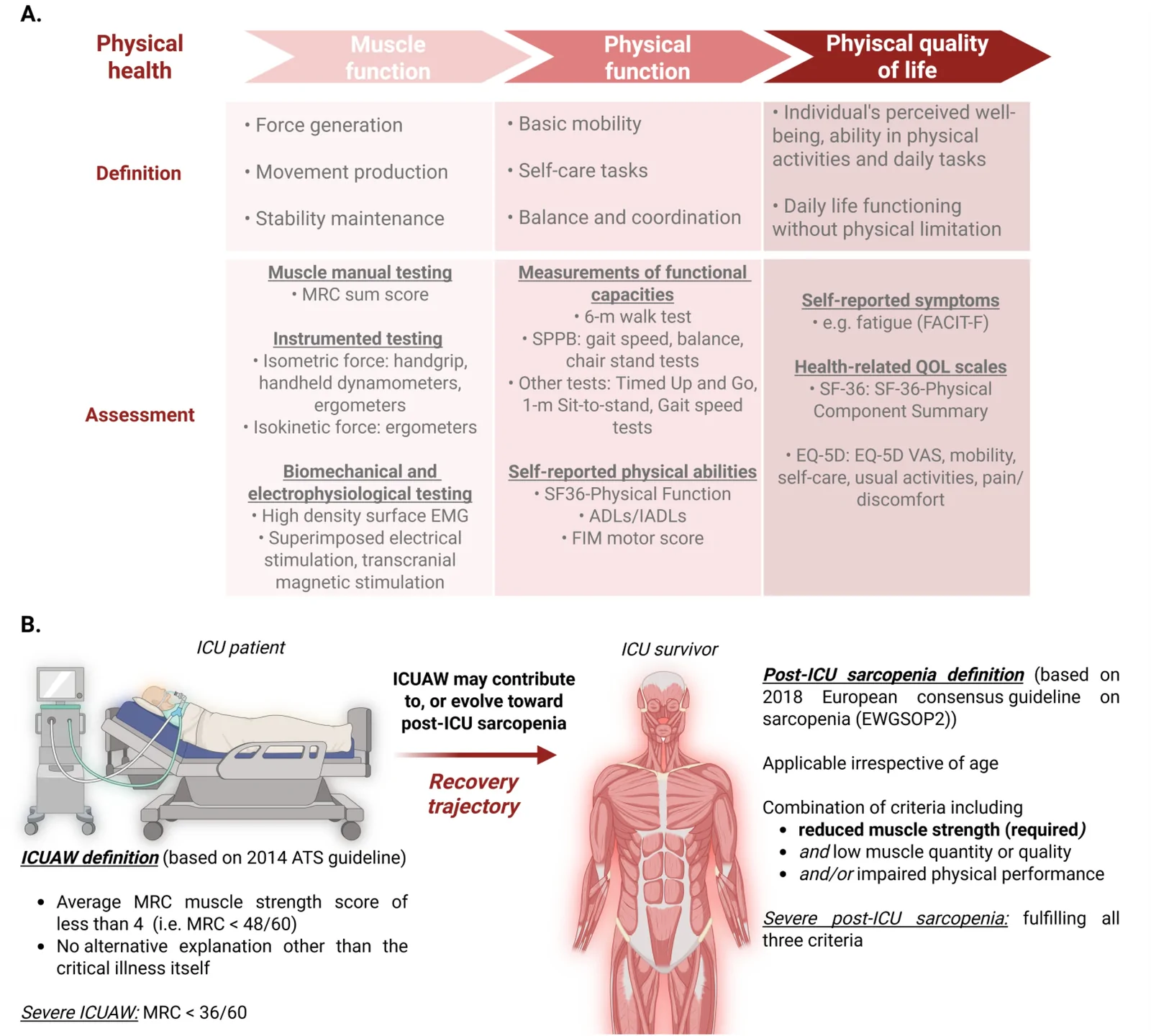
Conceptual framework for the assessment of physical health after critical illness and continuum from ICU-acquired weakness to post-ICU sarcopenia. A. Three interrelated domains: muscle function, physical function, and physical health-related quality of life. B. Continuum linking ICUAW to post-ICU sarcopenia during recovery. SF-36, Short Form Health Survey 36; FIM, Functional Independence Measure; ADLs, Activities of Daily Living; ICUAW, ICU-acquired weakness, IADLs, Instrumental Activities of Daily Living; FACIT-F, Functional Assessment of Chronic Illness Therapy–Fatigue; PCS, Physical Component Summary; EQ-5D, EuroQol 5-Dimension.

A comprehensive evaluation is essential to capture the impact of critical illness on functional and muscle recovery. It should rely on validated post-ICU assessment integrating standardized and patient-relevant outcomes to quantify deficits, stratify risk, and establish a robust baseline for targeted recovery strategies. The PRACTICE international Delphi study recently proposed a core outcome set for trials of physical rehabilitation after critical illness [26]. Physical health can be conceptualized through three interrelated domains: muscle function, physical function, and health-related quality of life (Fig. 2).

**(1) Muscle function**. Muscle force generation is most commonly assessed by manual muscle testing, quantified using the Medical Research Council (MRC) sum score. Instrumented testing (handheld dynamometers, ergometers) allows more precise evaluation of specific muscle groups [27], and high-density surface electromyography (EMG) provides additional mechanistic insight in advanced settings [27]. The diagnosis of ICUAW is based on manual muscle testing according to the American Thoracic Society guidelines [28], with an MRC sum score <48/60 after exclusion of alternative causes; no consensual definition exists for post-ICU muscle weakness. The MRC sum score fails to identify patients with persistent weakness during convalescence: in Dos Santos et al., all survivors had MRC > 48 six months after discharge but still showed reduced ergometric strength versus predicted values [29], and Van Aerde et al. reported a median MRC of 60/60 at five years despite persistent deficits in dynamometric strength, aerobic capacity, physical function and quality of life versus age/sex-matched controls [16]. Manual muscle testing is therefore insufficient to detect post-ICU muscle weakness, and complementary objective assessments are required for long-term follow-up. Rather than a strict diagnostic definition, the 2018 European sarcopenia consensus (EWGSOP2) offers a useful conceptual scaffold — combining reduced strength, low muscle quantity or quality, and impaired physical performance — for structuring long-term assessment [30] (Fig. 2). ICUAW and post-ICU sarcopenia nevertheless remain distinct entities: persistent ICUAW is best understood as a condition that may contribute to, or evolve toward, post-ICU sarcopenia during convalescence, rather than as synonymous with it. The MRC sum score nevertheless remains crucial during ICU stay and at discharge: a value <48 is associated with prolonged mechanical ventilation and ICU stay, increased hospital mortality, and long-term outcomes [5,31]. Values <55 are also associated with higher long-term mortality and disability compared with values above this threshold [8], and MRC scores at ICU discharge correlate with long-term strength, physical function and quality of life up to five years later [8].

Applying these criteria is, however, constrained by what can actually be measured, and the constraint differs markedly between the ICU stay and convalescence. During critical illness, assessment of muscle quantity and quality is particularly problematic: computed tomography (CT) and magnetic resonance imaging (MRI) — the reference modalities — require patient transport and are in practice available only opportunistically, while dual-energy X-ray absorptiometry (BXA) and bioelectrical impedance (BIA) analysis are confounded by the large fluid shifts of critical illness [32]. Point-of-care ultrasound is the only truly bedside modality and reliably tracks longitudinal muscle-thickness loss, but it remains limited by methodological heterogeneity — probe pressure, measurement site and operator dependence — by the absence of validated diagnostic thresholds, and by oedema, which inflates thickness while altering echogenicity independently of muscle content [33,34]. Estimates of muscle quantity and quality obtained during the acute phase should therefore be interpreted as within-patient trends rather than as diagnostic values. Beyond muscle quantity, muscle quality — captured by intramuscular adipose infiltration (myosteatosis), reduced CT radiodensity or increased ultrasound echogenicity — is increasingly recognised as an independent, prognostically relevant dimension in ICU survivors [[35], [36], [37]], although its bedside assessment shares the validation limitations noted above.

The situation differs after ICU discharge. Once fluid overload and acute inflammation have resolved, the survivor’s phenotype converges with that of the sarcopenic patient, and the consensus-validated toolbox may apply: muscle strength by handgrip dynamometry or the five-time chair-stand test; muscle quantity as appendicular skeletal muscle mass by DXA or BIA, with CT at the third lumbar vertebra and MRI as reference methods; muscle quality through CT radiodensity, ultrasound echogenicity or MRI fat fraction; and physical performance by gait speed, the Short Physical Performance Battery or the Timed Up and Go [30]. These frameworks continue to evolve: the Global Leadership Initiative in Sarcopenia now regards physical performance as an outcome of sarcopenia rather than one of its components [38], whereas the Sarcopenia Definition and Outcomes Consortium has argued for grip strength and gait speed in preference to DXA-derived lean mass [39]. It is on this basis that the sarcopenia framework becomes transposable to convalescence. For longitudinal follow-up, handgrip dynamometry provides a simple quantitative measure; the EWGSOP2 thresholds for probable sarcopenia (grip strength <27 kg in men and <16 kg in women) are commonly applied, complementing the MRC sum-score thresholds used during the ICU stay (<48 for the diagnosis of ICUAW; <55 as a prognostic threshold). These cut-offs were largely derived in non-ICU populations, and their diagnostic performance during post-ICU convalescence remains to be validated.

**(2) Physical function**. Capacity to perform daily activities (basic mobility, self-care, balance, coordination) is assessed through (a) standardized functional capacity measures — 6-minute walk test, Short Physical Performance Battery (SPPB), Timed Up and Go — and (b) patient-reported assessments in real-life contexts — Short Form Health Survey 36 (SF-36) Physical Function subscale, Functional Independence Measure (FIM) motor score, Activities of Daily Living (ADLs), and Instrumental Activities of Daily Living (IADLs).

**(3) Health-related quality of life**. Impaired physical function contributes to reduced health-related quality of life, commonly assessed with self-reported measures — Functional Assessment of Chronic Illness Therapy–Fatigue (FACIT-F), Physical Component Summary (PCS) of the SF-36, and EuroQol 5-Dimension (EQ-5D). Integrating these three domains provides a holistic understanding of post-ICU physical health and informs tailored rehabilitation strategies.

### Swallowing function

Swallowing function is an under-recognised dimension of physical recovery. Clinically significant post-extubation dysphagia affects approximately 30% of mechanically ventilated patients and can persist for months to years in a subset of survivors. Its multifactorial pathophysiology — laryngo-pharyngeal injury, pharyngeal muscle weakness and impaired central swallowing control — makes it both a manifestation of the wider neuromuscular deficit and an independent cause of malnutrition, aspiration, prolonged hospitalization, delayed functional recovery and increased 28- and 90-day mortality [40,41]. It therefore warrants systematic screening after extubation, with instrumental evaluation — fibreoptic endoscopic or videofluoroscopy — when screening is abnormal or oral intake remains insufficient.

## Metabolic, nutrition and physical rehabilitation

**Fig. 3:**
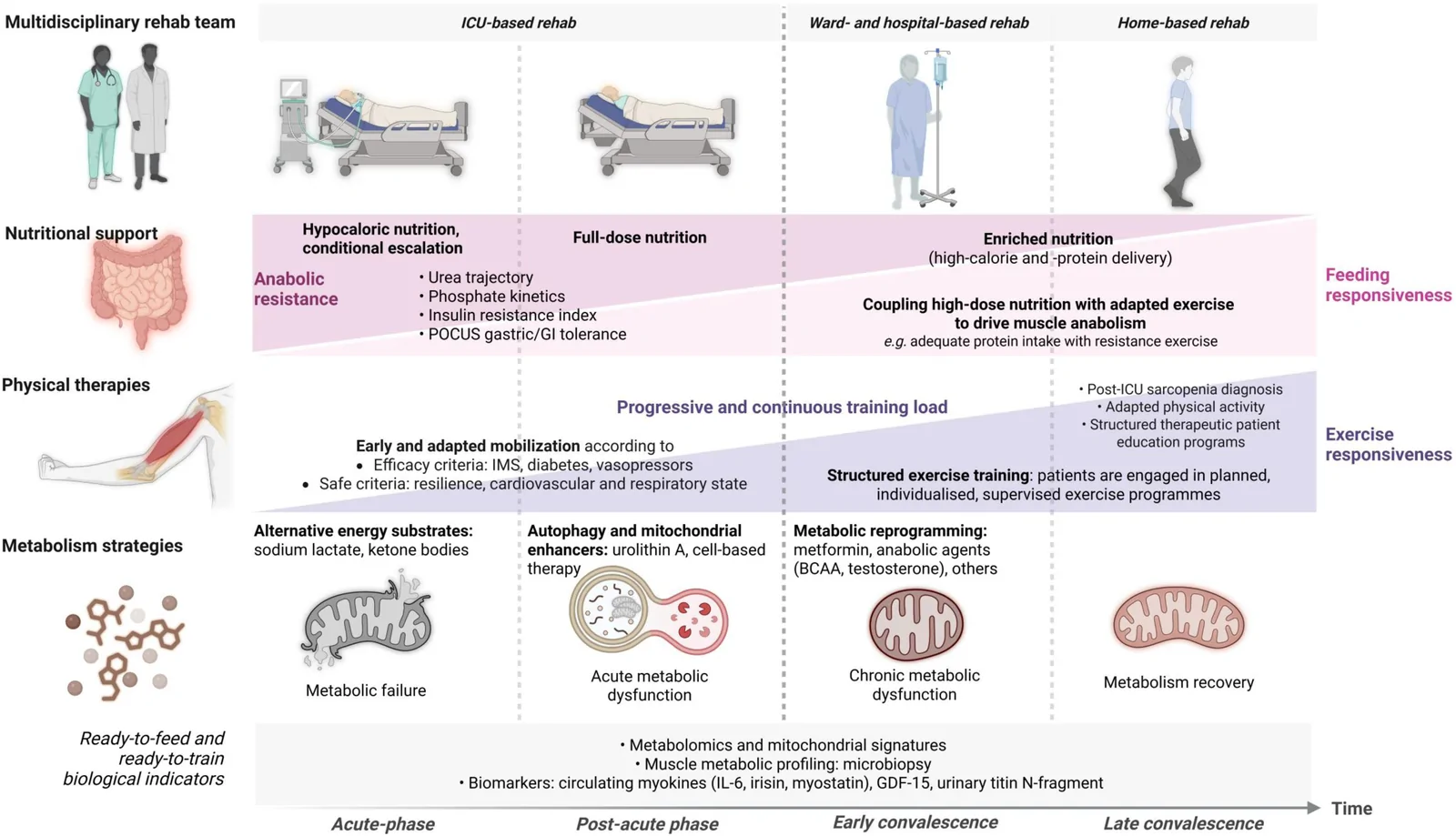
Integration of nutritional, physical and emerging metabolic strategies coupled to the biological transitions of skeletal muscle in critical illness. The figure synthesizes the proposed individualized care across four clinical phases and integrates three therapeutic dimensions. Mitochondrial morphology illustrates the conceptual transition from acute bioenergetic dysfunction to full metabolic recovery. Bedside-feasible biomarkers guide nutritional escalation; muscle metabolic profiling via metabolomics, mitochondrial signatures and microbiopsy remains a research perspective. The two gradient bands represent the biological continuum from anabolic resistance to feeding responsiveness (top) and from limited to full exercise responsiveness (bottom). BCAA, branched chain amino acids; IMS, ICU Mobility Scale; POCUS, point-of-care ultrasound; GI, gastrointestinal.

The persistence of muscle and functional alterations years after critical illness [1,24,25] underscores that recovery is not merely resolution of acute injury but reflects a pathobiology extending beyond ICU discharge. In the absence of any pharmacological strategy of proven clinical benefit for post-ICU muscle weakness, nutrition and physical therapy remain the two operational pillars of recovery. Yet, although physical rehabilitation improves short-term outcomes — muscle strength at ICU discharge, duration of mechanical ventilation and length of stay — multidisciplinary rehabilitation protocols have so far shown inconsistent effects on long-term physical outcomes [[42], [43], [44], [45], [46]]. Because short-term endpoints have been extensively addressed elsewhere, this review focuses on the long-term horizon of functional and muscle recovery. Understanding the clinical and biological determinants of these results is the prerequisite for designing more effective strategies. We apply this perspective in parallel to nutritional support and physical rehabilitation — reviewing for each the clinical evidence, the concept of responsiveness, and a phase-specific individualized strategy — and complement this framework with emerging metabolic support strategies (Fig. 3, Table 1).

**Table 1:** Nutritional and metabolic strategies across the phases of critical illness and recovery: current practice and research perspectives. EWGSOP2, European Working Group on Sarcopenia in Older People (2018); POCUS, point-of-care ultrasound.

	Metabolic response	Muscle response	Suggested current nutrition	Physical rehabilitation	Research perspectives
**Early acute phase** (first 48 hours)	• Metabolic sideration	• Acute mitochondrial dysfunction: bioenergetic failure	• Avoiding severe hyperglycaemia (target below 180 mg/dl) • No or minimal calorie supply	• Safety-first approach: minimise deep sedation; passive mobilisation and positioning as tolerated; avoid high-intensity mobilisation in haemodynamically unstable patients	• Bypassing conventional glucose oxidation pathways by leveraging alternative energy substrates: lactate sodium, ketones bodies
**Acute phase** (first 7 days)**, chronic critical illness** (persistent acute organ dysfunction after day 7)	• Anabolic resistance and increased catabolism; heterogeneous feeding responsiveness	• Progressive loss of muscle mass and force • Acute mitochondrial dysfunction and inflammation (immune cell infiltration)	• Glycaemia: as above • Restricting calorie and protein supply (6−8 kcal/kg/day and 0.2−0.4 g/kg/day) then cautious stepwise escalation • Individualization of nutrition support: monitoring (urea trajectory, phosphatemia, insulin resistance, gastric ultrasound) guides timing of escalation • Achieving standard targets (25 kcal/kg/day and 1 g/kg/day) in chronic critical illness	• Individualised early mobilisation titrated to physiological tolerance (right patient, right dose, right time); progression along the ICU Mobility Scale; NMES in selected patients; avoid prolonged low-intensity or excessive early mobilisation in vulnerable subgroups	• Biological profiling (e.g. muscle microbiopsy): monitoring guides timing of nutritional escalation (identification of the timing of the anabolic responsiveness shift) • Bypassing conventional glucose oxidation pathways by leveraging alternative energy substrates: ketogenic diet • Exploring feeding modalities that promote fasting-like metabolic responses: fasting mimicking diet, intermittent enteral nutrition • Autophagy and mitochondrial modulators: urolithin A
**Early convalescence** (resolution of acute organ dysfunction)	• Progressive resolution of anabolic resistance	• Progressive recovery of muscle mass and force • Progressive resolution of acute mitochondrial dysfunction and inflammation	• Glycaemia: as above • Increasing calorie and protein supply: at least 25 kcal/kg/day and 1−1.3 g/kg/day • Identifying frail and malnourished patients at high risk of underfeeding: continuing enteral nutrition • Identifying and managing post-extubation dysphagia	• Progressive active mobilisation and structured, supervised resistance and endurance training as tolerance improves; begin a comprehensive rehabilitation programme continued after discharge	• Alternative feeding modalities: ketogenic diet, fasting mimicking diet, intermittent enteral nutrition • Metabolic reprograming: metformin • Autophagy and mitochondrial modulators: urolithin A • Follow-up with protocol-guided and individualised nutritional support by dietitians
**Late convalescence** (after ICU discharge)	• Complete resolution of anabolic resistance	• Muscle mass may completely recover, but reduced strength may persist • Post-ICU sarcopenia (EWGSOP2 criteria) and physical health • Chronic mitochondrial dysfunction and inflammation • Stem cell dysfunction: impaired muscle regeneration • Extracellular matrix dysregulation: fibrosis	• Identifying and managing post-ICU insulin resistance and diabetes • Increasing calorie and protein supply: 30 kcal/kg/day and 1.5 g/kg/day	• Home- and community-based adapted physical activity; structured, progressive exercise training sustaining a continuous stimulus (specificity, overload, progression)	• Alternative feeding modalities: ketogenic diet, fasting mimicking diet • Metabolic reprograming: metformin • Autophagy and mitochondrial modulators: urolithin A • Follow-up with protocol-guided and individualised nutritional support by dietitians

### Nutritional support

#### Avoiding severe hyperglycaemia

Hyperglycaemia is a documented risk factor for ICUAW [31,47,48]. Severe hyperglycaemia experimentally disrupts neuronal and muscle homeostasis by inducing mitochondrial and autophagy dysfunction [[49], [50], [51], [52]]; insulin suppresses autophagy, but excess tissue glucose also impedes it [53]. Whether hyperglycaemia is pathogenic or adaptive remains debated [54]. The Leuven II study supported tight glucose control in patients on full parenteral nutrition (PN) for improving physical and mitochondrial outcomes [[55], [56], [57]]; the NICE-SUGAR trial showed that maintaining blood glucose <180 mg/dL reduces mortality and hypoglycaemia but does not improve recovery [58]. The recent TGC-fast trial, in patients not receiving full PN, found no benefit of tight (80–110 mg/dL) versus liberal (<215 mg/dL) glucose control [59]. The main difference between TGC-fast and Leuven II is the greater hyperglycaemia in Leuven II, where full PN — now known to be harmful due to overfeeding — likely worsened muscle metabolism, an effect mitigated by intensive insulin. Severe acute-phase hyperglycaemia therefore has a negative impact on long-term physical prognosis and should be avoided; the 2024 SCCM guidelines recommend tolerating moderate hyperglycaemia (<180 mg/dL) before introducing insulin [60].

#### Calorie and protein escalation: clinical evidence

Over the past 15 years, evidence has converged on a counter-intuitive conclusion: in the acute phase, more is not better, and in identifiable subgroups more is harmful. This contradicts observational data that identified the first-week cumulative calorie deficit as a risk factor for muscle weakness and prolonged convalescence [[61], [62], [63]], while experimental studies show that energy deficit does not worsen — and may improve — the muscle phenotype in ICU animals [51,[64], [65], [66]]. The reconciliation lies in acute-phase anabolic resistance (see Understanding feeding responsiveness) and in the protective role of fasting-induced repair pathways such as autophagy and ketogenesis [1,[67], [68], [69], [70], [71], [72], [73], [74], [75]].

##### Avoiding high energy intake

Large RCTs testing low-energy versus standard-energy feeding consistently found no benefit from increasing acute-phase calorie delivery (Table S2a). The clearest demonstration of harm from early calorie excess remains EPaNIC, where early PN prolonged ICU and ventilation duration, increased muscle weakness, and delayed recovery [66,76], likely through aggravation of autophagy dysfunction (autophagic markers inversely correlate with muscle weakness) [66,76]. The long-term EPaNIC follow-up confirmed the safety of withholding early PN [77]. EDEN (n = 1,000 ALI patients) compared trophic (∼400 kcal/d, ∼10 g protein/d) versus full enteral feeding for 6 days, with no difference in ventilator-free days or 60-day mortality but more gastrointestinal intolerance with full feeding [78]. PERMIT compared permissive underfeeding (∼835 kcal/d) versus standard feeding (∼1,300 kcal/d) for up to 14 days at similar protein delivery (∼0.7 g/kg/d), with no difference in 90-day mortality or functional outcomes [79,80]. TARGET (n = 3,957 ventilated patients) compared energy-dense (1.5 kcal/mL) versus routine enteral nutrition; despite ∼30% higher calorie delivery, 90-day mortality and patient-centered outcomes were unchanged [81,82]. NUTRIREA-3 extended this picture to the most severely ill: in 3,036 patients with shock and ventilation, low-dose feeding (6 kcal/kg/d, 0.2–0.4 g/kg/d) accelerated readiness for ICU discharge and reduced gastrointestinal complications without increasing 90-day mortality [83,84]. A post hoc analysis confirmed renal safety even in patients with preexisting renal dysfunction, with lower peak urea — consistent with the ureagenesis-mediated harm of high-dose protein documented in RE-EFFORT (see below) [84,85]. An important caveat applies to all of these trials: energy targets were defined from predictive equations or weight-based formulae (kcal/kg), with theoretical adjustments in patients with overweight or obesity. Indirect calorimetry has since shown that such estimates frequently misclassify true resting energy expenditure in individual patients, so that the delivered proportion of measured requirements — and hence the real degree of over- or under-feeding — may differ from the nominal targets.

##### Avoiding high protein intake

Evidence on protein dosing has been substantially refined over the past three years (Table S2b). EFFORT Protein randomized 1,301 patients at high nutritional risk to high (≥2.2 g/kg/d) versus usual (≤1.2 g/kg/d) protein for up to 28 days (achieved 1.6 vs 0.9 g/kg/d); the higher dose did not improve time to discharge at 60 days and signalled harm in patients with acute kidney injury (AKI) and higher SOFA scores [86]. The pre-specified AKI subgroup analysis confirmed an adjusted HR 1.39 for 60-day mortality among 311 baseline-AKI patients [87]. PRECISe extended these findings to patient-reported outcomes: in 935 ventilated patients randomized to 2.0 g/kg/d versus 1.3 g/kg/d for 28 days (achieved 1.9 vs 1.2 g/kg/d), the high-protein strategy worsened EQ-5D-5 L quality of life at 30, 90 and 180 days, with a clinically meaningful decrement [88]. A pre-specified Bayesian re-analysis of PRECISe showed a low probability of benefit and a high probability of increased 60-day mortality [89]. TARGET Protein consolidated the picture in unselected patients: 3,397 ventilated patients randomized to enteral protein-augmented (∼1.12 g/kg IBW/d achieved) versus standard formula (∼0.69 g/kg IBW/d) showed no benefit on days alive and out of hospital at 90 days; urea was again higher in the augmented group at day 5, and heterogeneity of treatment effect favoured usual protein in ventilated patients and in those developing new renal replacement therapy [90]. These findings are supported by meta-analyses [91,92]. A common feature — and limitation — of these trials is that the intervention was confined to the ICU period and delivered as a fixed target rather than a progressive, phase-adapted regimen; they therefore establish that early fixed-dose escalation is not beneficial, and may be harmful, but leave open whether a graded escalation matched to metabolic readiness would perform differently. The convergent results raise a central question: why does the supply of energy and amino acids that sustains anabolism in health fail — and at higher doses harm — the critically ill patient?

#### Understanding feeding responsiveness

##### Catabolic-to-anabolic transition window

Skeletal muscle physiologically requires sufficient amino acid and energy supply to sustain protein synthesis, regenerate, and recover its contractile capacity [93]. In critical illness, this anabolic capacity is markedly attenuated, and the central issue of acute-phase nutrition lies in the temporal mismatch between nutrient delivery and the muscle's ability to use it — operationalized as feeding unresponsiveness. Using stable-isotope tracer methodology, Chapple et al. showed that postprandial myofibrillar protein synthesis after enteral protein is ∼60% lower in ventilated ICU patients than in matched healthy controls, despite preserved digestion and amino acid absorption [94]. In a recent RCT, doubling the intraduodenal protein dose (40 vs 20 g) failed to further augment postprandial muscle protein synthesis despite higher plasma leucine availability [95], confirming that anabolic resistance reflects an intrinsic muscle defect that cannot be overcome by dose escalation. Combined with sustained proteolysis, this blunted anabolic response unifies the mechanism of acute-phase muscle wasting [93] and the lack of benefit of early nutritional escalation *per se*. Anabolic resistance does not persist throughout the ICU stay: clinical and experimental data suggest a transition window during which feeding responsiveness is regained, paralleling resolution of inflammation and recovery of mitochondrial function and insulin sensitivity [1]. Identifying this window in real time is the central unmet need — feeding too early is futile and potentially deleterious, while delaying escalation beyond it may exacerbate cumulative deficits that themselves contribute to muscle wasting. The duration of anabolic resistance is highly heterogeneous and not adequately captured by calendar-based recommendations [96,97].

##### Dynamic biological indicators

Several biological signatures of anabolic resistance have recently been proposed as candidate readouts of feeding responsiveness. These signatures should be interpreted with caution: they derive predominantly from secondary analyses or single-cohort modelling studies, and none has been prospectively validated as a marker of feeding readiness. In RE-EFFORT, Haines et al. showed that median serum urea rose 2.1 mmol/L higher in the high-protein group by day 6, and that the excess mortality with high-dose protein was mediated by an upward urea trajectory, with a hazard ratio of 1.34 per twofold rise in serum urea after adjustment for age, illness severity, AKI, and renal replacement therapy [85]. Serum urea and the urea-to-creatinine ratio therefore represent simple biochemical markers of impaired protein utilization and increased ureagenesis, and may identify patients in whom protein delivery should be delayed [98,99]. These markers are, however, non-specific: dehydration, gastrointestinal bleeding, trauma, corticosteroid therapy and renal dysfunction may all raise urea independently of protein utilisation. Lauwers et al. reanalysed EPaNIC and showed that an early phosphate decrease (>0.16 mmol/L over the first 2 ICU days) — but not absolute hypophosphataemia — interacted with treatment: in patients decreasing phosphatemia, early PN prolonged ICU dependency (adjusted HR 0.75 for earlier discharge), whereas no significant harm was seen otherwise [100]. Phosphate trajectory may thus serve as an accessible dynamic indicator of unreadiness for full feeding. More recently, Gargi et al. developed a trajectory-based model integrating an insulin resistance index with haemodynamic and inflammatory criteria in 2,350 ICU patients; the catabolic-to-anabolic transition occurred in 94% of patients (∼60% by day 3) with substantial inter-individual variability [101]. High caloric delivery (≥1.0 kcal/kg/h for ≥24 h) before this transition was independently associated with increased 90-day mortality in a dose-dependent fashion, whereas calendar-based escalation failed to discriminate outcomes. Dynamic biological indicators are therefore more discriminative than fixed calendar criteria for capturing the transition from anabolic resistance to feeding responsiveness, but it should currently be considered a plausible hypothesis requiring prospective validation, not a validated bedside test.

##### Muscle metabolic profiling

While these bedside indicators provide accessible signals of metabolic (un)readiness, they remain indirect surrogates of the muscle metabolic state, and direct profiling of the muscle biological phenotype is a complementary translational strategy. By measuring autophagy flux in human primary muscle cells exposed to ICU patient serum, Tardif et al. identified three distinct metabolic phenotypes — ICU non-responders, inducers, and blockers — that may underpin individual feeding responsiveness [102]. Once standardized, such a test could be obtained within ∼3 days and may carry significant implications for personalizing nutritional support. Directly mapping muscle metabolic processes in vivo would represent an important step toward biology-guided nutritional therapy. Mini-invasive microbiopsy provides ∼10 mg of tissue, with less pain and discomfort than a Bergström biopsy and feasibility even in patients at haemorrhagic risk; this is sufficient for focused metabolic or miRNA signatures [103]. Looking forward, integrating bedside-feasible biomarkers (urea trajectory, phosphate kinetics, insulin resistance index) with mechanistic readouts from microbiopsy (autophagy flux, mitochondrial signatures) into composite ready-to-feed indicators — prospectively validated against patient-centred functional outcomes — is a key research priority to move from calendar-based toward truly individualized nutritional support [68].

#### Proposed individualized nutrition strategy

**Fig. 4:**
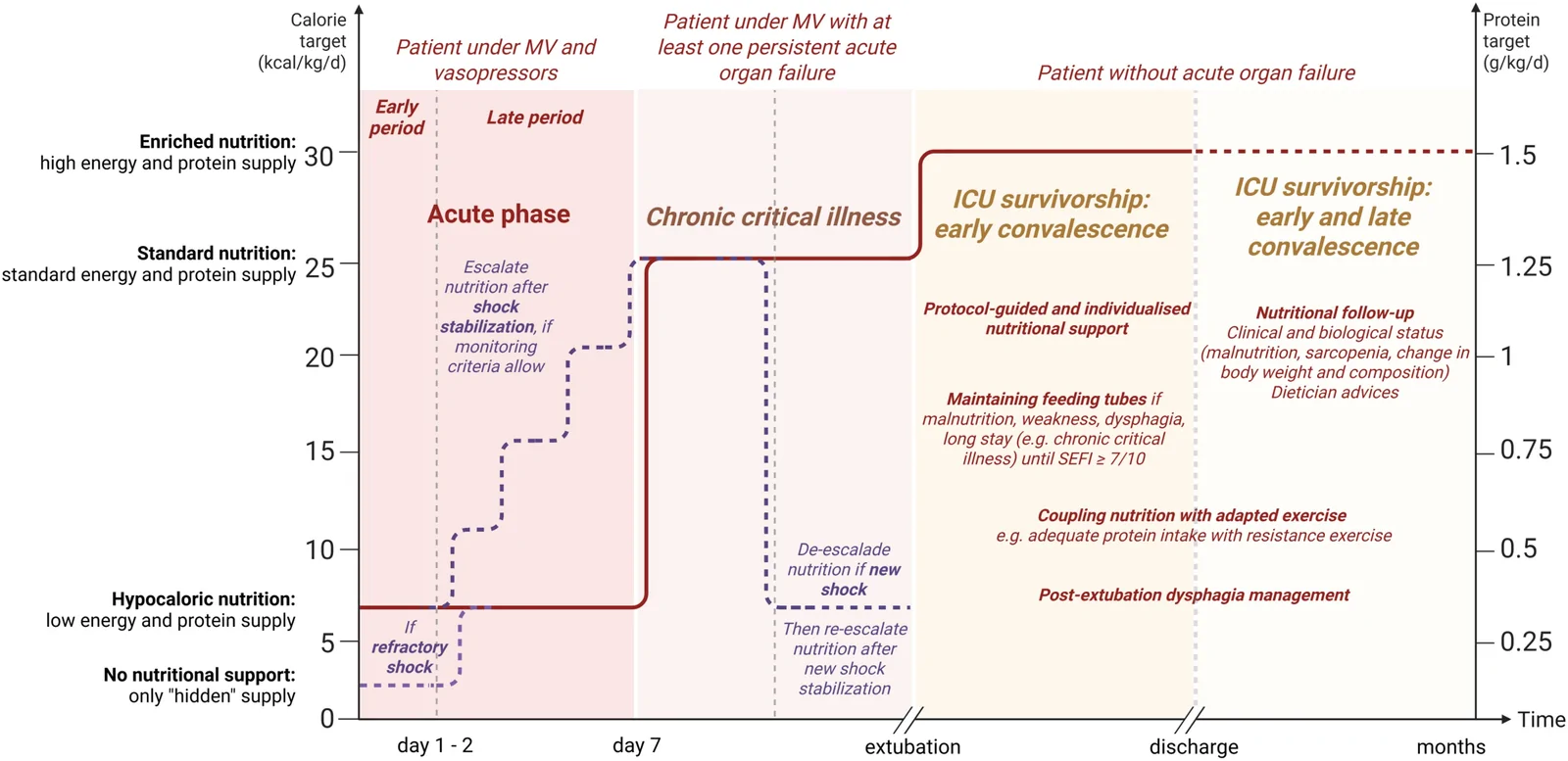
Proposed phase-specific individualized strategy for nutritional strategy. Nutritional support across four temporal phases is represented on a dual Y-axis (weight-based calorie target, kcal/kg/d, left; protein target, g/kg/d, right). Where indirect calorimetry is available, measured resting energy expenditure (mREE) could be used in place of the weight-based calorie target, with tentative equivalences of approximately 30% of mREE during hypocaloric nutrition, 80–100% at standard targets and 110–125% during late convalescence. These correspondences are indicative and have not been prospectively evaluated. The bold red line indicates the guideline-aligned nutritional trajectory; the dashed purple line indicates conditional escalation or de-escalation guided by monitoring criteria (achieved calorie and protein delivery relative to target; urea trajectory, phosphate kinetics, insulin resistance index, gastrointestinal tolerance). MV, mechanical ventilation; SEFI, Self-Evaluation of Food Intake.

Translating these mechanistic insights into a pragmatic bedside strategy requires reconciling current guidelines with the evidence reviewed above. The 2023 ESPEN guideline recommends setting energy targets from indirect calorimetry, with hypocaloric feeding in the early acute phase (≤70% of measured energy expenditure during the first three days, or below 70% of estimated needs when indirect calorimetry is unavailable), progressive escalation toward 80–100% thereafter, and a progressive protein target of 1.3 g/kg/d [104]. The 2025 SRLF guidelines, informed by recent RCTs, push permissive underfeeding further: 6–8 kcal/kg/d (BMI-adjusted) and 0.2–0.9 g/kg/d during the first ICU week in ventilated patients, returning to standard targets (20–30 kcal/kg/d, 1.0–1.3 g/kg/d) thereafter [105]. Building on these guidelines and the concept of feeding responsiveness, we propose a phase-specific individualized nutrition strategy (Fig. 4) articulated around four clinical periods mirroring the transition from anabolic resistance to feeding responsiveness, designed to be coupled with the parallel evolution from limited to full exercise responsiveness detailed in Tailored physical interventions.

***(1a) Acute phase, early period (day 1–2)****: permissive underfeeding or "hidden" supply.* In ventilated patients on vasopressors, hypocaloric, low-protein nutrition should be initiated within 48 h, targeting 6–8 kcal/kg/d and 0.2–0.4 g/kg/d. In refractory shock, only "hidden" caloric supply (drug carriers, glucose from buffered solutions, citrate from regional anticoagulation) should be provided.

***(1b) Acute phase, late period (day 3–7)****: conditional escalation.* Escalation toward standard targets should be progressive and conditional on metabolic readiness, guided by the dynamic indicators above: an upward urea trajectory or sustained urea-to-creatinine rise should delay protein escalation; an early relative phosphate decrease (>0.16 mmol/L over 2 days) should prompt energy restriction and phosphate repletion; a persistently elevated insulin resistance index identifies patients still in catabolism. Enteral feeding should also be deferred in repeated vomiting, regurgitation, abdominal distension, or progressively rising antral cross-sectional area on POCUS, which correlates with aspirated gastric volume and predicts feeding intolerance and 28-day mortality [106,107]. Absent these signals, calorie and protein delivery can be progressively titrated up to 20 kcal/kg/d and 1.0 g/kg/d.

***(2) Chronic critical illness (day 7 to extubation)****: standard targets with reactive de-escalation.* Patients with at least one persistent acute organ failure should receive standard nutrition (20–25 kcal/kg/d, 1.0–1.3 g/kg/d). A new shock episode triggers temporary de-escalation back to permissive underfeeding, with re-escalation only after haemodynamic stabilization — reflecting the dynamic course of ICU recovery and that the transition window can be transiently lost.

***(3) ICU survivorship*** *— early convalescence (extubation to ICU/hospital discharge): protocol-guided, individualized support.* Once acute organ failure has resolved, nutritional therapy may shift from harm avoidance toward active anabolic support, although the evidence base remains limited. Pragmatic targets of 1.2–1.5 g/kg/d of protein combined with structured resistance exercise can be proposed in line with sarcopenia guidelines [30], but no RCT has demonstrated long-term functional benefit. A tailored nutrition intervention initiated in the ICU and continued throughout hospitalisation is feasible and increases energy delivery (INTENT phase II RCT: +271 kcal/day versus usual care), although effects on patient-centred outcomes remain to be tested in adequately powered trials [108]. Feeding tubes can reasonably be maintained in patients with persistent malnutrition, ICU-acquired weakness, post-extubation dysphagia or chronic critical illness, until oral intake recovers (Self-Evaluation of Food Intake [SEFI] score ≥7/10 as a clinical surrogate of adequate spontaneous intake [109]). Oral intake after ICU discharge is generally insufficient, particularly when enteral nutrition was discontinued prematurely [[110], [111], [112], [113]]; supplemental enteral nutrition was associated with the highest calorie intake, although reaching dietician-calculated targets did not translate into improved functional recovery [113]. Protocol-guided, individualized nutritional support by specialist dieticians has reduced mortality in non-critically ill medical inpatients at nutritional risk [114]; whether this can be extrapolated to ICU survivors is plausible but unproven. Dysphagia is the main barrier to the resumption of oral nutrition and should be managed within the nutritional pathway rather than separately. Speech therapist evaluation, texture and viscosity modification, compensatory postural manoeuvres and targeted swallowing exercises form the mainstay. Pharyngeal electrical stimulation is the instrumental option: it accelerated readiness for decannulation in tracheotomised post-stroke patients (49% versus 9% with sham) [115], but evidence in general ICU populations remains limited to pilot studies. As for limb NMES, its effect presumably depends on preserved neural integrity, and dedicated trials in ICU survivors are needed.

***(4) Late convalescence (post-discharge to months)****: enriched nutrition with structured follow-up.* After hospital discharge, achieving energy and protein targets is generally more challenging. Expert recommendations propose enriched intakes of 30–35 kcal/kg/d and 1.2–1.5 g/kg/d, with high-protein oral nutrition supplements, combined with home-based adapted physical activity (APA) and structured therapeutic patient education [116]. However, data on actual energy needs in survivors are scarce, indirect calorimetry being demanding in spontaneously breathing patients [111]. Using a canopy hood, Ridley et al. and Rousseau et al. reported a measured energy expenditure ∼23 kcal/kg/d in ICU survivors [111,117], with a daily energy deficit averaging ∼5% of measured expenditure in the Ridley study — although measurements were single-time and in a selected subset, suggesting that survivors are no longer hypermetabolic in long-term recovery. Whether high calorie targets are warranted remains unresolved. Even when energy balance and muscle mass are restored, weakness frequently persists [64,118,119]. Nutritional approaches alone are unlikely to reverse the sustained molecular and cellular alterations of critical illness. Nutrition must be paired with progressive, individualized exercise and with emerging biological strategies targeting energy homeostasis and cellular machinery.

### Physical rehabilitation

#### Managing physical therapy: clinical evidence

##### Within the ICU

Physical therapy in ventilated patients has been extensively studied over the past two decades, yet — as for nutrition — more is not necessarily better, and current evidence supports a shift from dose-based to physiology-informed prescription (Table S3). The principal mobilization and rehabilitation trials are summarised in the supplementary material (Table S4). Studies of protocolized early and progressive mobilization show favourable short-term effects on muscle strength at ICU discharge, ventilation duration, and length of stay, but these benefits are transient and do not consistently translate into long-term functional recovery [[120], [121], [122], [123], [124], [125], [126], [127], [128], [129]]. Early mobilization is generally safe, although harmful effects can occur [127,128], and specific risk-assessment tools have been developed for patients receiving vasoactive drugs [130].

The TEAM trial provided the pivotal demonstration of this dose-related ceiling effect [131]. In 750 ventilated patients across 49 ICUs in 6 countries, augmented-intensity early mobilization (median 21 vs 9 min. d of active mobilization) failed to improve days alive and out of hospital at 180 days, while increasing adverse events. Three pre-specified or post hoc secondary analyses reframed the negative result by showing who was harmed and why. First, the diabetes-stratified analysis identified an interaction signal in 21% of participants: augmented-dose mobilization more than tripled 180-day mortality in diabetic patients [132]. Second, the target trial emulation tested 25 prespecified combinations of duration and intensity (ICU Mobility Scale, IMS) and showed that prolonged mobilization was associated with increased 28-day mortality when patients only achieved low intensity (≥20 min at IMS 2), but that this harm signal disappeared once IMS ≥ 4 (standing or above) was reached [133]. Third, the individualized treatment effects analysis (machine-learning causal inference) confirmed marked between-patient heterogeneity in the benefit-versus-harm balance, suggesting the null average result masks both responders and patients harmed by high-dose mobilization [134].

Functional and adjunctive interventions have not closed this gap. In-bed cycling and Functional Electrical Stimulation in-bed Cycle Ergometry (FESCE), Neuromuscular Electrical Stimulation (NMES), and exercise training in addition to usual care have been shown safe but not effective on muscle strength, mass, or short- and long-term physical outcomes [129,[135], [136], [137], [138], [139]]. Patients at highest risk of prolonged ICU stay but with lower illness severity may benefit most [126], and Wu et al. recently showed that resistance training in selected patients with low illness severity who could perform the exercises improved muscle strength and physical functioning at hospital discharge [140].

Current guidelines recommend initiating early mobilization, « adapted to the patient's resilience and general condition », within the first ICU days, delivered by a multidisciplinary team with protocolized safety criteria [45,141]. Yet the recent evidence calls for a clear paradigm shift: the goal is no longer « providing more mobilization » but « providing the right mobilization, to the right patient, at the right dose, and at the right time » [142] — a shift from fixed-dose protocols toward physiology-informed, adaptive prescription integrating pre-ICU functional status, illness severity, comorbidities (diabetes, frailty, sarcopenia), real-time tolerance, and the patient's evolving capacity to respond. This concept of inter-individual responsiveness is developed in the next section.

##### After the ICU

Beyond ICU discharge, rehabilitation may be delivered through ward-based interventions, dedicated rehabilitation units, or at-home outpatient programmes. The evidence base remains limited, with few RCTs published. Walsh et al. conducted the first RCT of combined physical and nutritional rehabilitation initiated in the post-ICU hospital ward, with a rehabilitation practitioner coordinating therapy, dietetic management and educational support; the intervention did not improve any physical recovery outcome [42] but improved patient experience and perceived quality of care [143]. Gruther et al. assessed an intensive ward-based programme (mobilization, resistance/aerobic exercise, NMES) delivered 2 h/d, 5 d/week until discharge — six times usual care duration (∼20 min/d) — without superior physical recovery [144], although per-protocol analysis suggested shorter length of stay. Several structural reasons may account for this absence of long-term benefit. Duration may be insufficient: both trials ended at hospital discharge, whereas exercise-induced adaptations require sustained and progressive stimulus [145,146]. The transition between hospital- and home-based rehabilitation remains a documented care gap, with most patients losing access to structured therapy after discharge. The delivery of sustained post-ICU rehabilitation is constrained by tangible structural barriers — limited physiotherapy staffing and equipment, uneven access to specialist rehabilitation units, variable reimbursement, and the well-documented discontinuity at the hospital-to-home transition — that operate in both acute and community settings [147]. These implementation barriers, as much as any biological ceiling, must be addressed for any physiology-informed strategy to be deliverable at scale. Remote delivery may partly circumvent these constraints.

More recently, the iRehab trial randomised 429 survivors of mechanical ventilation to a six-week remote multicomponent programme — targeted exercise, symptom management, psychological and peer support — initiated within twelve weeks of hospital discharge [148]. The primary outcome, health-related quality of life at eight weeks, did not improve significantly (EQ-5D-5 L utility 0.69 versus 0.67). The intervention nevertheless improved four of six secondary outcomes, including leg strength and exercise capacity as well as fatigue and anxiety. This dissociation is informative in itself: the programme moved the muscle-function and physical-function domains without shifting a generic preference-based utility index, which argues for the multidimensional assessment outlined above rather than for a purely negative reading. In a stepped-wedge cluster randomised trial conducted in twenty Brazilian public hospitals, an integrated telehealth programme spanning the whole trajectory — an ICU component targeting ventilator liberation, a ward component providing risk stratification and individualised rehabilitation planning, and a two-month personalised post-discharge telerehabilitation programme — improved health-related quality of life at ninety days after discharge in 1,916 adults with acute hypoxaemic respiratory failure (EQ-5D-3 L utility 0.16 versus 0.12), and also shortened mechanical ventilation (9.9 versus 15.5 days) and reduced ninety-day mortality (71.8% versus 78.3%) [149]. The utility gain was not observed among survivors (0.60 versus 0.59), indicating that it was driven largely by the mortality difference, and the very high baseline mortality of this resource-constrained setting limits extrapolation. The trial nonetheless shows that a strategy deployed continuously across the ICU, the ward and the home can shift hard clinical endpoints, whereas programmes confined to a single phase have not — an argument for continuity and phase-appropriateness of the stimulus rather than for intensity within any one period.

Larger RCTs evaluating physical therapy beyond hospital discharge are therefore warranted. Adapted physical activity (APA) programmes — effective in several chronic disorders [150,151] — represent an attractive avenue, allowing continuity of care, increased cumulative dose, and individualization. The ongoing NUTRIREA-4 trial (NCT06581939) is assessing the combined effect of optimized post-ICU nutrition and online APA-based rehabilitation in survivors.

#### Understanding exercise responsiveness

##### Inter-individual heterogeneity of response

Exercise improves physical performance through multiple cellular and systemic networks [152,153] and exerts anti-inflammatory and metabolic effects across muscle disuse, sarcopenia and cachexia [[154], [155], [156]]. Yet a substantial proportion of ICU patients remain exercise non-responders. This phenomenon — absence of expected adaptive gains in strength, mass or metabolic health following structured training — has been documented in elderly populations [157], in patients with type 2 diabetes [158,159], and in sport science [160]. Determinants involve endogenous (genetics, epigenetics, transcriptome, metabolome) and exogenous (intensity, duration, modality, nutrition, sleep) factors [161,162]. Critical illness is now emerging as a paradigmatic state of exercise non-responsiveness.

##### Clinical signatures of non-response

The clinical heterogeneity of exercise response in critical illness has been explored in secondary and post hoc analyses of the TEAM trial, which suggest three candidate correlates of non-response — pre-existing diabetes, inability to reach upright posture (IMS < 4), and high illness severity (high-dose vasopressors, RASS ≤ −3). These signals come from a single trial and remain unvalidated; they may inform bedside mobilization decisions but should not be used in isolation to guide them.

A further, largely unexplored source of heterogeneity is the neuromuscular phenotype itself. No rehabilitation trial has stratified patients by CIM, CIP or mixed forms, and outside critical care the two lesions call for different emphases: resistance training in inflammatory myopathies [163], versus sensorimotor and balance retraining paced by axonal regeneration in acquired polyneuropathies [164]. One corollary is directly testable: conventional NMES depolarises motor axons and so requires an intact lower motor neuron, leaving denervated fibres unrecruited by standard parameters [165]. Trials of NMES and FESCE in unselected populations may therefore have averaged a real effect in innervated patients with its absence in those with axonal loss — a concrete stratification hypothesis for future trials.

##### Molecular determinants of non-response

The biological substrate of this clinical signal is beginning to be characterized. In a recent negative RCT of FESCE in ventilated patients, RNA sequencing of the electrically stimulated muscle revealed persistent inflammatory activation (NF-κB, IL-6/TNF-α) and downregulation of mitochondrial biogenesis and oxidative phosphorylation genes, providing a transcriptomic explanation for the absence of benefit despite an effective mechanical stimulus [137,166]. These data support the hypothesis that ICU survivors remain weak not only because of atrophy but because of molecular abnormalities including unresolved inflammation, mitochondrial dysfunction, altered satellite-cell function, and disturbed proteostasis [167]. Whether bedside biomarkers — circulating myokines (IL-6, irisin, myostatin), GDF-15, urinary titin N-fragment — could serve as dynamic readouts of exercise readiness [[168], [169], [170], [171]], in parallel with the indicators developed in Understanding feeding responsiveness, is a key research priority.

##### Toward personalized exercise prescription

Mapping the molecular dynamics of exercise response in critical illness will require dedicated translational programmes. The NIH MoTrPAC consortium has begun to deliver such a multi-omic atlas of exercise adaptation in health and disease [172], and analogous initiatives are needed in the ICU population, possibly integrating microbiopsy-derived profiling (autophagy and mitochondrial signatures, miRNA panels) with the clinical and biological signatures above. Such a framework would move the field from population-average toward truly personalized exercise prescription.

#### Proposed tailored physical interventions

Translating this evidence into a pragmatic bedside strategy requires shifting from dose-based to physiology-informed mobilization. Building on current guidelines [45,141], we propose a phase-specific tailored strategy (Fig. 3) articulated around four periods coupled with the parallel nutritional strategy.

***(1) Acute phase (day 1–7)****: mobilization with safety-first prescription.* In ventilated patients on vasopressors, mobilization should be limited to passive range of motion, in-bed exercises and upright positioning (IMS 0–3), with a safety threshold informed by validated tools for haemodynamic, respiratory and neurological resilience [130]. Prolonged sessions targeting higher IMS levels should be deferred in patients unable to achieve IMS ≥ 4 (standing) [133]. Caution also applies to diabetes, frailty, advanced age or high illness severity, in whom physiological reserves are likely reduced — a hypothesis warranting dedicated investigation. Mobilization should be deferred in deeply sedated patients (RASS ≤ −3) and in those on high-dose vasopressors, in line with validated expert "traffic light" frameworks [173].

***(2) Chronic critical illness (persistent organ failure ≥ 7 days)****: conditional escalation guided by exercise readiness.* In patients with persistent acute organ failure but who can tolerate upright posture (IMS ≥ 4), progressive escalation toward standing and transfer to chair (IMS 4–6) is reasonable, with duration matched to tolerance (rating of perceived exertion, heart-rate and ventilatory response). A new shock episode or biological signal of metabolic intolerance (rising lactate, deteriorating urea) triggers temporary de-escalation.

***(3) ICU survivorship — early convalescence (extubation to hospital discharge)****: structured, supervised exercise programme.* Physical therapy may shift from harm avoidance toward active anabolic stimulation. The patient should enter a planned, individualized, supervised exercise programme designed to extend continuously from ICU discharge to home-based convalescence, rather than isolated rehabilitation episodes, in cooperation with a multidisciplinary team [174]. Beyond the physiotherapist, this team integrates dieticians (for adequate protein and energy intake), speech therapists (for dysphagia management), occupational therapists (for daily-living function), and exercise specialists (for training prescription). This shift is grounded in three principles of exercise training: specificity (modality targeting molecular/functional deficits — resistance for sarcopenia, aerobic for mitochondrial dysfunction, balance for proprioceptive deficits), overload (training stress exceeding habitual load), and progression (gradual systematic load increase to sustain continuous adaptation) [175]. Selected patients with low illness severity who can perform the exercises may benefit most from resistance training [140].

***(4) Late convalescence (post-discharge to months)****: continuity of the training stimulus through home- and community-based programmes.* After discharge, the central challenge is maintaining the training continuum and accumulating a sufficient cumulative dose — likely the missing ingredient in past trials, which uniformly ended at hospital discharge. Home-based APA programmes — combining structured sessions (resistance, aerobic, balance), therapeutic patient education, online APA platforms, wearable devices — represent the most promising avenue, allowing individualization of frequency, intensity, type and time-course and continuous application of specificity, overload and progression. Although robust evidence is awaited (NUTRIREA-4 ongoing), this phase is integral to the recovery trajectory, not optional.

*Future perspectives.* The phase-specific strategy outlined here remains pragmatic; the next step is true individualization. Integrating clinical signatures of non-response (e.g. diabetes, IMS) with dynamic biological readouts (e.g. myokines) and mechanistic readouts from muscle microbiopsy (e.g. mitochondrial profiling) into composite ready-to-train indicators, validated against patient-centred functional outcomes [26], will be required for precision medicine and tailored exercise (Table S3).

### Emerging metabolic support

#### Utilizing alternative energy substrates

##### Utilizing lactate

Long viewed as a passive end-product of anaerobic glycolysis and a biomarker of tissue hypoperfusion, lactate is increasingly recognized as a central metabolic intermediate and candidate therapeutic agent in critical illness [68,176]. Beyond its role as the metabolizable anion of balanced crystalloids, hypertonic sodium lactate combines volume expansion with metabolic and signalling effects [177]. By providing a metabolizable anion paired with sodium without chloride load, it avoids hyperchloraemic acidosis and produces a positive haemodynamic effect with smaller infused volumes. Its rationale as an alternative energy substrate rests on three properties. First, lactate is taken up via monocarboxylate transporters (MCT1/MCT4) in an insulin-independent manner, bypassing acute-phase tissue insulin resistance. Second, it enters mitochondrial oxidation without ATP-consuming activation, with a higher ATP-to-oxygen yield than free fatty acids. Third, lactate participates in inter-organ shuttles redistributing carbon and reducing equivalents from glycolytic to oxidative tissues — heart, brain, kidney and liver acting as net consumers under stress [178,179] — providing the basis for substrate-based protection of vital organs in the acute phase. Consistent with this rationale, exogenous lactate exerts protective effects in experimental shock [[180], [181], [182], [183], [184], [185]], improved cardiac performance in pilot human trials in acute heart failure [186], and is currently being evaluated in sepsis (NCT03528213). Beyond its role as a fuel, lactate exerts pleiotropic signalling effects including dampening of NLRP3 inflammasome activation, histone lactylation reprogramming toward reparative phenotypes, and direct mitochondrial action [187,188]. Lactate-based therapy thus offers an attractive framework for the acute phase, when conventional macronutrient delivery is futile or harmful.

##### Utilizing ketone bodies

Ketone bodies serve as alternative energy substrates during low glucose availability such as prolonged fasting [189]. Ketogenesis from fatty acids starts within 12–24 h of fasting in humans [190]. Ketogenic diets attenuate inflammation, support mitochondrial metabolism, and induce autophagy [191,192]. Ketogenesis is impaired in critical illness, and exogenous ketone body supplementation has been shown to improve outcomes, particularly muscle-related ones. Experimentally, exogenous ketones prevent sepsis-induced muscle weakness [193,194], and exogenous ketone esters enhance cardiac contractility in cardiogenic shock [195]. Inhibition of ketogenesis has been suggested to explain the harmful effects of full feeding in paediatric and adult critically ill patients [69,70]. Ketogenic feeding has recently been proposed as a safe alternative to standard enteral nutrition for bioenergetic failure in critical illness [71,72], and allows complete insulin withdrawal. In satellite cells isolated from patients with ICU-acquired weakness, exposure to β-hydroxybutyrate restored complex II activity and lowered reactive oxygen species production [196], directly linking ketone availability to the regenerative compartment discussed below. However, the current evidence base for ketogenic strategies is preliminary: it rests largely on experimental sepsis models and on small pilot or early-phase human studies with short follow-up and surrogate endpoints. Overall, ketone body treatment — by diet, enteral feeding, or exogenous administration — holds promise for long-term muscle weakness by alleviating mitochondrial and autophagy dysfunction. Whether ketone bodies improve long-term physical outcomes remains to be explored.

#### Promoting autophagy and mitochondrial health

Nutrients influence autophagy: amino acids suppress it more than glucose or lipids [73,74]. Feeding-induced suppression can be reversed by autophagy inducers in critically ill animals [74]. Nutritional interventions enhancing autophagy in the ICU — such as fasting-mimicking diets [197,198] — remain investigational. In post-ICU patients, no nutritional intervention is specifically designed to induce autophagy. Rapamycin, an autophagy inducer that is also immunosuppressive, is difficult to manage. Urolithin A, a natural postbiotic, holds promise for addressing long-term muscle weakness by promoting autophagy flux [119,199,200]. Metabolic reprogramming through repurposed agents represents another emerging avenue. Metformin, through AMPK activation and modulation of mitochondrial complex I and autophagy, holds promise for supporting post-ICU muscle and functional recovery [[201], [202], [203], [204], [205], [206]].

Beyond autophagy, the mitochondrion is itself a therapeutic target, and the translational landscape divides into two families [68]. Antioxidant strategies aim to neutralise mitochondrial oxidative stress; despite consistent preclinical protection they have not improved clinical outcomes, and high-dose vitamin C increased death or persistent organ dysfunction in septic patients [68]. Non-antioxidant approaches instead target mitochondrial integrity, dynamics and quality control and appear more promising for muscle, although none has been evaluated in ICU survivors [68]. The recurring obstacle is translational rather than mechanistic: dose, timing and disease stage determine preclinical efficacy but are rarely reproduced in trials, and human mitochondrial phenotyping remains too coarse to identify who might respond [68].

#### Promoting anabolism

If acute-phase anabolic resistance explains why supplying substrate fails, then pharmacologically forcing anabolism during that window should fail as well — and may cause harm. Recombinant growth hormone provides the definitive demonstration: in two parallel randomised trials in prolonged critical illness it approximately doubled in-hospital mortality [207], and a recent meta-analysis confirms that growth hormone and anabolic steroids improve nitrogen balance without improving survival, while prolonging ICU and hospital stay [208]. Androgens make the mirror-image point: in severe burns, where catabolism persists beyond the acute inflammatory phase, testosterone reduces muscle protein breakdown [209] and its synthetic analogue oxandrolone improves net protein balance and lean mass [210], gains amplified by concurrent exercise. Selective androgen receptor modulators remain untested here, and a trial of early transdermal testosterone in critically ill patients is ongoing (NCT05825092). The sex-specific dimension is essentially unexplored: oestradiol deficiency depletes the satellite cell pool and impairs strength recovery after repeated injury [211], and transdermal oestrogen augments the muscle-mass gain from resistance training in early postmenopausal women [212], yet no study has asked whether female survivors — particularly postmenopausal ones — require a different anabolic strategy. Amino acids act at the same node: leucine activates mTORC1 through the Sestrin–GATOR2 axis, and its metabolite β-hydroxy-β-methylbutyrate (HMB) — partly converted to HMG-CoA, the precursor shared by cholesterol synthesis and ketogenesis — has been evaluated as a muscle-preserving supplement [[213], [214], [215]]. Anabolic resistance blunts precisely this step, which is consistent with the failure of branched-chain amino acids and their metabolites, HMB included, to preserve muscle during the acute phase of critical illness [215]. These agents therefore share a plausible but untested window — the convalescent phase, once anabolic responsiveness returns — and should be evaluated in combination with a training stimulus rather than as a substitute for one.

#### Others pharmacological strategies

Two further avenues target the cellular substrate of impaired regeneration rather than metabolism. Critical illness activates senescence programmes irrespective of age — beneficial early, by conferring resistance to apoptosis, but deleterious later through a pro-inflammatory and pro-fibrotic secretory phenotype; senolytics and senomorphics are therefore candidate therapies, conditional on defining the therapeutic window and monitoring senescent burden [216]. Muscle regeneration itself depends on satellite cells, which are quantitatively or qualitatively impaired after critical illness: their content is reduced six months after discharge in survivors with sustained atrophy [29], and even when the pool is numerically preserved, proliferative and myogenic capacity remain compromised [217]. This makes the regenerative compartment a therapeutic target in its own right: engrafting mesenchymal stem cells restores satellite cell function and muscle strength in septic mice [218], but cell-based therapy in ICU survivors remains entirely preclinical.

Across these strategies the pattern is consistent: a strong mechanistic rationale, convergent preclinical protection, and an almost complete absence of clinical validation in survivors (Table S5).

## Conclusion

Functional and muscle recovery after critical illness remains an unmet clinical priority despite gains in short-term survival. The convergent failure of randomized trials to demonstrate long-term benefit from intensified nutritional or rehabilitative interventions — despite the short-term gains that early mobilization does achieve — has clarified that the critically ill and convalescent muscle is not the muscle of a healthy individual recovering from acute injury, but a tissue with persistent biological alterations that reshape its response to feeding and exercise stimuli — a concept we have operationalized as feeding and exercise responsiveness. The corollary is that effective interventions must be matched to biological readiness rather than calendar day, and that combining nutritional support, physical rehabilitation and emerging metabolic support — within and beyond the ICU stay — is more likely to outperform any single intervention delivered in isolation.

The phase-specific individualized strategies outlined in this review remain pragmatic and population-based (Table S6); the next step is true biology-guided individualization, through composite ready-to-feed and ready-to-train indicators integrating bedside-feasible biomarkers with mechanistic readouts from muscle microbiopsy, prospectively validated against patient-centred functional outcomes. Adaptive platform trials randomizing patients on the basis of such biomarkers are a logical next step. The muscle of the critically ill patient is an integrative target whose recovery depends on the synchronized restoration of feeding and exercise responsiveness across the full trajectory from ICU admission to home-based convalescence.

## Authors' contributions

A.P. wrote the main manuscript, and all authors provided substantial review and revisions.

## Consent for publication

Not applicable.

## Ethics approval and consent to participate

Not applicable.

## Funding

The study was supported by the French National Research Agency (ANR-23-CE14-0063-01).

## Availability of data and materials

Not applicable.

## Declaration of competing interest

The authors declare that they have no competing interests.
