# Supplementary material for: Functional and muscle recovery after critical illness: current and future nutritional, physical and metabolic strategies
Source: Ann Intensive Care. 2026 Aug 24;16:100137. doi: 10.1016/j.aicoj.2026.100137 (PMC13571473; doi:10.1016/j.aicoj.2026.100137)
Supplement: Supplementary file 1 [file mmc1.docx]

| **Term** | **Definition** | **Assessment and thresholds** |
| --- | --- | --- |
| **ICU-acquired weakness (ICUAW)** | Symmetrical, diffuse limb weakness developing during critical illness, with no cause other than the illness itself | MRC sum score <48/60 (ATS criteria), after adequate awakening  Handgrip dynamometry as an adjunct |
| **Critical illness myopathy (CIM)** | Primary myopathy: reduced myofibre excitability, preferential myosin loss and altered contractility | Electrophysiology and/or muscle biopsy  Predominant driver of sustained force loss in survivors |
| **Critical illness polyneuropathy (CIP)** | Acute, length-dependent, axonal sensorimotor polyneuropathy | Nerve conduction studies  Associated with slower and less complete neurological recovery |
| **Post-ICU sarcopenia** | Persistent low muscle strength with low muscle quantity or quality and impaired physical performance during convalescence | Framed conceptually on EWGSOP2  Distinct from ICUAW, which may contribute to or evolve toward it |
| **Muscle function** | Capacity to generate force | MRC sum score  Handheld and handgrip dynamometry  High-density surface EMG |
| **Physical function** | Capacity to perform daily activities: mobility, self-care, balance and coordination | 6MWT, SPPB, Timed Up and Go  FIM, ADL and IADL scales |
| **Health-related quality of life** | Perceived physical well-being and ability to engage in daily life | FACIT-F  SF-36 Physical Component Summary  EQ-5D |
| **Physical and functional recovery** | Recovery of muscle force and structure, and of the ability to perform daily activities and social roles | Longitudinal, multidimensional assessment |
| **Post-intensive care syndrome (PICS)** | New or worsening physical, cognitive and/or mental health impairment persisting after critical illness | Multidomain follow-up |

**Table S1.** Definitions of the main terms used throughout the manuscript. ADL: activities of daily living; ATS: American Thoracic Society; EMG: electromyography; EQ-5D: EuroQol 5-Dimension; EWGSOP2: European Working Group on Sarcopenia in Older People 2018; FACIT-F: Functional Assessment of Chronic Illness Therapy–Fatigue; FIM: Functional Independence Measure; IADL: instrumental activities of daily living; ICU: intensive care unit; MRC: Medical Research Council; SF-36: Short Form Health Survey 36; SPPB: Short Physical Performance Battery; 6MWT: six-minute walk test.

| **Trial** | **Population** | **Intervention vs control (delivered)** | **Primary outcome** | **Main result** | **Take-home** |
| --- | --- | --- | --- | --- | --- |
| **EPaNIC**  Casaer *et al.* NEJM 2011 (1)  Casaer *et al.* ICM 2024 (2) | 4,640 mixed ICU patients nutritionally at risk (7 Belgium ICUs) | Early PN (within 48 h): PN to reach 100% target (~25–30 kcal/kg IBW/d, ~0.8–1.1 g/kg/d protein)  *vs* Late PN (start ≥ day 8): EN only (delivery ~50% target, ~0.2–0.4 g/kg/d protein) for first week | Composite: ICU LOS; new infection | Late-PN: shorter ICU stay (HR 1.06), fewer infections, less ICU-acquired weakness  2-yr FU: no difference in mortality, Barthel, EQ-5D | Early PN harms; permissive underfeeding is safe long-term |
| **EDEN**  Rice *et al.* JAMA 2012 (3) | 1,000 patients with ALI/ARDS on MV (44 US ICUs) | Trophic EN ~400 kcal/d, ~10 g protein/d × 6 d  *vs* Full EN ~1300 kcal/d, ~50 g protein/d | Ventilator-free days at d28 | No difference in VFD, 60-d mortality, or infections; more GI intolerance with full feeding | Trophic feeding improves GI tolerance in ARDS |
| **PERMIT**  Arabi *et al.* NEJM 2015 (4) | 894 mixed ICU patients on MV (7 Saudi Arabia and Canada ICUs) | Permissive underfeeding 40–60% kcal (~835 kcal/d)  *vs* Standard 70–100% (~1,300 kcal/d)  Similar protein delivery (~0.7 g/kg/d) in both arms | 90-d all-cause mortality | No difference in mortality, ICU/hospital LOS, or functional outcomes | Permissive underfeeding (calorie restriction with preserved protein) is safe |
| **TARGET**  Chapman *et al.* NEJM 2018 (5) | 3,957 patients on MV (46 ANZ ICUs) | Energy-dense EN 1.5 kcal/mL (~1863 kcal/d) x 28 d  *vs* Routine EN 1.0 kcal/mL (~1262 kcal/d)  ≈ 30% higher kcal in intervention arm  Similar protein delivery (~1.0 g/kg IBW/d; ~55 g/l) in both arms | 90-d all-cause mortality | No difference in mortality, MV duration, or infections | Augmenting calorie density does not help |
| **NUTRIREA-3**  Reignier *et al.* Lancet Respir Med 2023 (6) | 3,036 patients on MV with shock (61 French ICUs) | Low-dose 6 kcal/kg/d, 0.2–0.4 g protein/kg/d × 7 d  *vs* Standard 25 kcal/kg/d, 1.0–1.3 g protein/kg/d | Co-primary: time to ICU-discharge readiness; 90-d mortality | Faster readiness for ICU discharge (HR 1.12); fewer GI events (vomiting, diarrhea, bowel ischaemia, liver dysfunction)  No difference in 90-d mortality | In shock + MV, low-dose feeding is superior on recovery and neutral on mortality in shock |

**Table S2a.** Summary of the main randomized controlled trials of acute-phase energy delivery in critically ill patients discussed in the section « Avoiding high energy intake ». This table is not a systematic review of all available trials but presents the studies cited and developed in the manuscript text. EN: enteral nutrition; PN: parenteral nutrition; MV: mechanical ventilation; LOS: length of stay; FU: follow-up; HR: hazard ratio; ALI/ARDS: acute lung injury / acute respiratory distress syndrome; IC: indirect calorimetry; GI: gastrointestinal; HRQoL: health-related quality of life; IBW: ideal body weight.

| **Trial** | **Population** | **Intervention vs control (delivered)** | **Primary outcome** | **Main result** | **Take-home** |
| --- | --- | --- | --- | --- | --- |
| **EFFORT Protein**  Heyland *et al.* Lancet 2023 (7) | 1,301 patients on MV nutritionally at risk (85 ICUs, 16 countries) | High protein ≥2.2 g/kg/d × 28 d  *vs* Usual ≤1.2 g/kg/d  Achieved 1.6 vs 0.9 g/kg/d  Similar energy delivery (~14 kcal/kg/d) | Time to discharge alive at d60 | No benefit; signal for harm in AKI and high-SOFA subgroups | High protein does not improve outcomes; harm in vulnerable subgroups |
| **EFFORT-AKI**  Stoppe *et al.* Crit Care 2023 (8) | 311 patients with baseline AKI (post-hoc EFFORT) | Same arms as EFFORT | 60-d mortality | Higher mortality with high protein in AKI: aHR 1.39 (1.03–1.87) | AKI identifies the subgroup most vulnerable to high-dose protein |
| **RE-EFFORT**  Haines *et al.* Crit Care 2024 (9) | EFFORT cohort — Bayesian joint mediation analysis | Same arms as EFFORT | Serum urea trajectory | High protein ↑ urea (+2.1 mmol/L at d6, +3.0 at d12)  Each 2-fold ↑ urea: HR 1.34 (1.21–1.48) for 30-d mortality | Ureagenesis = mechanism of harm; urea trajectory as bedside biomarker |
| **PRECISe**  Bels *et al.* Lancet 2024 (10) | 935 patients on MV (5 Dutch ICUs, 5 Belgian ICUs) | High 2.0 g/kg/d × 28 d  *vs* Standard 1.3 g/kg/d  Achieved 1.9 vs 1.2 g/kg/d  Similar energy delivery (~19 kcal/kg/d) | EQ-5D-5L HRQoL during 180 days | Worse HRQoL at d30, d90, d180 in high-protein arm | High protein impairs HRQoL |
| **PRECISe re-analysis**  Schouteden *et al.* Clin Nutr 2025 (11) | PRECISE cohort — pre-planned Bayesian analysis | Same arms as PRECISe | 60-d mortality | Low probability of benefit; high probability of ↑ 60-d mortality | Robust signal of mortality harm under Bayesian framework |
| **TARGET Protein**  Summers *et al.* JAMA 2025 (12) | 3,397 mechanically ventilated patients (8 ICUs ANZ) | Augmented EN 100 g/L formula  *vs* Standard 63 g/L; cross-over design  Achieved 1.12 vs 0.69 g/kg IBW/d  Similar energy delivery | Days alive and out of hospital at d90 | No benefit (median 62 vs 64 d, p=0.46)  ↑ urea at d5/d10; HTE favouring usual protein in MV (p=0.02) and new RRT (p<0.001) | Confirms absence of benefit and AKI/RRT-restricted harm |

**Table S2b.** Summary of the main randomized controlled trials of acute-phase protein delivery in critically ill patients discussed in the section « Avoiding high protein intake ». This table is not a systematic review of all available trials but presents the studies cited and developed in the manuscript text. AKI: acute kidney injury; RRT: renal replacement therapy; HTE: heterogeneity of treatment effect; ANZ: Australia and New Zealand; TSA: trial sequential analysis; aHR: adjusted hazard ratio.

|  | **Current studies** | **Research perspectives** |
| --- | --- | --- |
| **Population** | Less severe patients: ceiling effect — rapid recovery regardless of intervention  More severe patients: floor effect — intervention dose not effectively delivered or received  Heterogeneity: preexisting conditions and functional status (diabetes, sarcopenia), ICU conditions (ARDS or septic shock ≠ post-operative) | Identifying a similar phenotype: intermediate-risk patients may derive greater benefit than other subgroups?  Stratification by functional capacity (e.g. IMS ≥ 4) to identify subgroups likely to benefit |
| **Intervention** | Mobilization too early in some patients: no benefit or harm  Mobilization tailored to patient resilience and cardiovascular and respiratory status: safe criterion  Standalone intervention  No biological window of opportunity → no muscular adaptation  Insufficient cumulative dose and training stimulus (intensity, interval dosing, progressive overload, repetition): transient benefits | Heterogeneity of individual treatment effects: identifying responders vs non-responders  Mobilization integrated into a bundle (nutrition, co-medications, sedation)  Operationalizing exercise responsiveness through clinical signatures and molecular determinants  Better understanding of molecular mechanisms (anabolic resistance, inflammation, metabolic dysfunction) and testing novel targeted pharmacological approaches  Identifying the optimal exercise: type (resistance, endurance), dose (duration, intensity, frequency)  Increasing the cumulative dose: integrating mobilization into a comprehensive rehabilitation programme started at ICU admission and continued after discharge, including home-based rehabilitation  Structured exercise training: planned, individualised, supervised exercise programme (e.g. at least 12 weeks)  Respect of exercise training principles: specificity, overload, progression |
| **Control** | Modest separation in delivered dose (control group already receiving mobilization and exercise)  Threshold effect rather than a dose–response relationship? | Definition and standardisation of standard of care in multicentre studies, to increase exercise and sedation homogeneity and improve the likelihood of demonstrating a beneficial effect |
| **Outcome** | Mortality and short-term outcomes: not the objective? | Core outcome set for trials of physical rehabilitation  Physical performance and health-related quality of life months after ICU discharge: 6MWD, physical SF-36, EQ-5D-5L  Additional patient-centred outcomes: fatigue, activities of daily living, participation, return to work |

**Table S3.** Methodological limitations of current physical rehabilitation trials in critical illness and research perspectives, structured by the PICO framework (Population, Intervention, Control, Outcome). 6MWD: 6-minute walk distance; ARDS: acute respiratory distress syndrome; EQ-5D-5L: EuroQol 5-Dimension 5-level; ICU: intensive care unit; IMS: ICU Mobility Scale; PICO: Population–Intervention–Control–Outcome; SF-36: Short Form Health Survey 36.

| **Trial** | **Population** | **Intervention vs control** | **Primary outcome** | **Main result** | **Take-home** |
| --- | --- | --- | --- | --- | --- |
| **TEAM (NEJM 2022)** | 750 ventilated patients, 49 ICUs, 6 countries | Augmented early mobilization (median 21 vs 9 min/day) | Days alive and out of hospital at 180 days | No benefit; more adverse events | Higher dose is not better |
| **TEAM — diabetes analysis (AJRCCM 2024)** | Diabetic subgroup (21%) | High- vs usual-dose mobilization | 180-day mortality | Mortality more than tripled with the high dose | Harm concentrated in a subgroup |
| **TEAM — dose emulation (AJRCCM 2026)** | Target-trial emulation | 25 duration × intensity combinations | 28-day mortality | Prolonged low-intensity mobilization (IMS 2) increased mortality; resolves at IMS ≥4 | Intensity threshold rather than “more is better” |
| **RECOVER / Walsh (JAMA Intern Med 2015)** | 240 post-ICU ward patients | Increased ward rehabilitation and information vs usual care | Physical function (RMI) at 3 months | No improvement; better patient experience | Ward dose alone is insufficient |
| **Gruther (Am J Phys Med Rehabil 2017)** | Post-ICU ward patients | Intensive programme (~2 h/day) vs usual care | Length of stay and physical function | No superior physical recovery; per-protocol analysis suggested shorter LOS | Intensity alone is insufficient |
| **FESCE (Thorax 2021)** | Ventilated patients | FES cycle ergometry vs usual care | Muscle and functional outcomes | Safe but not effective | Adjunctive stimulation ineffective |
| **In-bed cycling (NEJM Evid 2024)** | Ventilated patients | Early in-bed cycling vs usual care | Physical function (SPPB) at discharge | No significant benefit | Cycling alone is insufficient |
| **Resistance training (Wu, Crit Care 2023)** | Medical ICU, four arms ± HMB | Resistance training vs usual care | Strength and physical function at discharge | Benefit in selected lower-severity patients | Selected patients may respond |
| **iRehab (JAMA 2026)** | 429 post-ICU survivors, MV ≥48 h, 52 UK hospitals | 6-week remote multicomponent rehabilitation vs standard care | EQ-5D-5L utility at 8 weeks | Primary endpoint not met (+0.04, below the MCID of 0.08); 4 of 6 secondary outcomes improved, including leg strength and exercise capacity | Gains in the muscle and physical domains missed by a generic utility index |
| **Tele-Rehab (JAMA 2026)** | 1,916 adults with acute hypoxaemic respiratory failure, 20 Brazilian public hospitals | Integrated ICU, ward and post-discharge telerehabilitation vs usual care | EQ-5D-3L utility at 90 days | Higher utility (+0.049); MV shortened by 6.2 days; mortality 71.8% vs 78.3%; no difference among survivors | Continuity across phases shifts hard endpoints; gain driven by mortality |
| **Meta-analysis (Carayannopoulos, Crit Care Med 2025)** | Enhanced vs usual mobilization RCTs | Pooled analysis | Function and adverse events | Benefits on strength, ventilation duration and LOS; heterogeneous, not uniformly negative | Individualized, well-dosed rehabilitation may benefit |

**Table S4.** Principal mobilization and rehabilitation trials in critical illness discussed in the manuscript. This table is not a systematic review of all available trials but presents the studies cited and developed in the manuscript text. AJRCCM: American Journal of Respiratory and Critical Care Medicine; EQ-5D: EuroQol 5-Dimension; FES: functional electrical stimulation; FESCE: functional electrical stimulation in-bed cycle ergometry; HMB: β-hydroxy-β-methylbutyrate; ICU: intensive care unit; IMS: ICU Mobility Scale; LOS: length of stay; MCID: minimal clinically important difference; MV: mechanical ventilation; RCT: randomized controlled trial; RMI: Rivermead Mobility Index; SPPB: Short Physical Performance Battery.

| **Agent or class** | **Mechanism** | **Evidence and setting** | **Phase-appropriateness** | **Translational status** |
| --- | --- | --- | --- | --- |
| **Sodium lactate** | Insulin-independent oxidative substrate; anti-inflammatory and mitochondrial signalling | Protective in experimental shock and cardiac arrest  Pilot human study in acute heart failure  Sepsis trial ongoing (NCT03528213) | Acute phase | Investigational |
| **Ketone bodies and ketogenic feeding** | Alternative oxidative substrate; autophagy and mitochondrial support; allows insulin withdrawal | Prevent sepsis-induced muscle weakness in rodents  Small pilot study and one open-label randomized trial; β-hydroxybutyrate restores complex II activity in satellite cells from ICU patients ex vivo | Acute phase to early convalescence | Investigational |
| **Fasting-mimicking and intermittent feeding** | Promotion of autophagy | Pilot crossover studies | Acute phase to chronic critical illness | Investigational |
| **Urolithin A** | Mitophagy activation and restoration of autophagic flux | Prevents long-term sepsis-induced muscle and mitochondrial dysfunction in mice  Safety data in healthy humans | Convalescence | Early-translational |
| **Metformin** | AMPK activation; complex I modulation; autophagy; anti-senescence | Improves sepsis-induced muscle weakness in mice  Favourable observational ICU data | Early convalescence | Early-translational |
| **Elamipretide (SS-31)** | Cardiolipin stabilization and protection of the mitochondrial inner membrane | Prevents post-sepsis chronic muscle weakness and sepsis-induced diaphragm dysfunction in rodents  No human data in critical illness | Acute phase to convalescence | Preclinical |
| **NAD⁺ precursors (nicotinamide riboside, nicotinamide mononucleotide)** | Restoration of NAD⁺ availability and sirtuin signalling | NAD⁺ depletion documented in ICU patients  Raise NAD⁺ in acute kidney injury and long COVID without clinical benefit | Convalescence | Early-translational |
| **Mitochondria-targeted antioxidants (MitoQ, coenzyme Q10, high-dose vitamin C)** | Scavenging of mitochondrial reactive oxygen species | Consistent preclinical organ protection  High-dose vitamin C increased death or persistent organ dysfunction in sepsis (LOVIT) | Not supported in the acute phase | Negative in randomized trials |
| **Recombinant growth hormone** | Stimulation of the growth hormone–IGF-1 axis | Approximately doubled mortality in prolonged critical illness  Meta-analysis: improved nitrogen balance, no survival benefit, longer ICU and hospital stay | Contraindicated in the acute phase | Harmful acutely |
| **Testosterone and oxandrolone** | Androgen receptor signalling; increased net protein balance | Reduce protein breakdown and improve lean mass in severe burns, amplified by exercise  No functional benefit in unselected critically ill patients  Transdermal testosterone trial ongoing (NCT05825092) | Convalescence (hypothesis) | Investigational |
| **Selective androgen receptor modulators** | Tissue-selective androgen receptor agonism | No data in critical illness | Convalescence (hypothesis) | Untested |
| **Oestrogens (women)** | Oestrogen receptor α signalling; satellite cell maintenance; enhanced response to training | Oestradiol deficiency depletes the satellite cell pool and impairs strength recovery in mice  Transdermal oestrogen augments training-induced muscle mass gain in early postmenopausal women | Convalescence (hypothesis) | Untested after ICU |
| **HMB, leucine and branched-chain amino acids** | mTORC1 activation; anabolic substrate provision | Systematic review: no acute-phase benefit on muscle mass, nitrogen balance or strength | Not for the acute phase; possibly post-ICU | Re-evaluation warranted |
| **Senolytics and senomorphics** | Elimination of senescent cells or suppression of the senescence-associated secretory phenotype | Senescence programmes activated in critical illness irrespective of age  Senolytics reduce organ injury in septic mice | Convalescence | Preclinical |
| **Mesenchymal stem cells** | Restoration of satellite cell regenerative capacity and of muscle repair | Satellite cells are quantitatively or qualitatively impaired in ICU patients and survivors; engraftment restores satellite cell function and muscle strength in septic mice | Convalescence | Preclinical |

**Table S5.** Emerging metabolic and pharmacological strategies discussed in the manuscript, with mechanism, current evidence, the recovery phase in which each is most plausibly relevant, and translational status. This table is not a systematic review of all available agents but presents the strategies cited and developed in the manuscript text. AMPK: AMP-activated protein kinase; HMB: β-hydroxy-β-methylbutyrate; ICU: intensive care unit; IGF-1: insulin-like growth factor 1; LOVIT: Lessening Organ Dysfunction with Vitamin C trial; mTORC1: mechanistic target of rapamycin complex 1; NAD: nicotinamide adenine dinucleotide.

| **Recovery phase** | **Nutritional evidence** | **Rehabilitation evidence** | **Metabolic and pharmacological** | **Level of evidence and objective** |
| --- | --- | --- | --- | --- |
| **Acute phase (day 1–2 to first week)** | EPaNIC, EDEN, PERMIT, TARGET, NUTRIREA-3  EFFORT Protein, PRECISe, TARGET Protein | TEAM and its secondary analyses (diabetes; dose and IMS)  Tele-Rehab (ICU component) | Sodium lactate; ketone bodies (preclinical and pilot) | Multiple large RCTs  Objective: avoid harm — permissive underfeeding, safety-first mobilization |
| **Chronic critical illness (persistent organ failure ≥7 days)** | Standard-target guidance, largely extrapolated | FESCE, in-bed cycling, NMES trials (neutral) | Fasting-mimicking and intermittent feeding (pilot) | Few RCTs  Objective: maintain, avoid harm, escalate conditionally |
| **Early convalescence (extubation to hospital discharge)** | RECOVER, Gruther, INTENT (feasibility, neutral) | RECOVER, Gruther (ward-based)  Resistance training in selected patients (Wu)  Tele-Rehab (ward component) | Metformin; urolithin A (early-translational) | Few, mostly neutral RCTs  Objective: begin active anabolic support |
| **Late convalescence (after hospital discharge)** | Dietitian-led individualized support, extrapolated from non-ICU populations | iRehab (remote multicomponent)  Tele-Rehab (telerehabilitation component)  Home-based and adapted physical activity programmes; NUTRIREA-4 (ongoing) | Combined home nutrition and adapted physical activity (ongoing) | Sparse evidence  Objective: restore function and accumulate cumulative training dose |

**Table S6.** Principal studies mapped onto the four phases of recovery. Nutritional randomized trials cluster in the acute and chronic critical illness phases, whereas rehabilitation trials span the whole trajectory; metabolic strategies remain largely preclinical or early-phase. Tele-Rehab is, to date, the only trial to have delivered a rehabilitation strategy across all four phases. FESCE: functional electrical stimulation in-bed cycle ergometry; ICU: intensive care unit; IMS: ICU Mobility Scale; NMES: neuromuscular electrical stimulation; RCT: randomized controlled trial.

References

1.	Casaer Michael P., Mesotten Dieter, Hermans Greet, Wouters Pieter J., Schetz Miet, Meyfroidt Geert, et al. Early versus Late Parenteral Nutrition in Critically Ill Adults. New England Journal of Medicine. 2011;365(6):506–17. doi:10.1056/NEJMoa1102662

2.	Casaer MP, Stragier H, Hermans G, Hendrickx A, Wouters PJ, Dubois J, et al. Impact of withholding early parenteral nutrition on 2-year mortality and functional outcome in critically ill adults. Intensive Care Med. 2024 Jul 17. doi:10.1007/s00134-024-07546-w

3.	National Heart, Lung, and Blood Institute Acute Respiratory Distress Syndrome (ARDS) Clinical Trials Network, Rice TW, Wheeler AP, Thompson BT, Steingrub J, Hite RD, et al. Initial trophic vs full enteral feeding in patients with acute lung injury: the EDEN randomized trial. JAMA. 2012 Feb 22;307(8):795–803. doi:10.1001/jama.2012.137 PubMed PMID: 22307571; PubMed Central PMCID: PMC3743415.

4.	Arabi Yaseen M., Aldawood Abdulaziz S., Haddad Samir H., Al-Dorzi Hasan M., Tamim Hani M., Jones Gwynne, et al. Permissive Underfeeding or Standard Enteral Feeding in Critically Ill Adults. New England Journal of Medicine. 2015;372(25):2398–408. doi:10.1056/NEJMoa1502826

5.	TARGET Investigators, for the ANZICS Clinical Trials Group, Chapman M, Peake SL, Bellomo R, Davies A, Deane A, et al. Energy-Dense versus Routine Enteral Nutrition in the Critically Ill. N Engl J Med. 2018 Nov 8;379(19):1823–34. doi:10.1056/NEJMoa1811687 PubMed PMID: 30346225.

6.	Reignier J, Plantefeve G, Mira JP, Argaud L, Asfar P, Aissaoui N, et al. Low versus standard calorie and protein feeding in ventilated adults with shock: a randomised, controlled, multicentre, open-label, parallel-group trial (NUTRIREA-3). The Lancet Respiratory Medicine. 2023 Mar 20;0(0). doi:10.1016/S2213-2600(23)00092-9

7.	Heyland DK, Patel J, Compher C, Rice TW, Bear DE, Lee ZY, et al. The effect of higher protein dosing in critically ill patients with high nutritional risk (EFFORT Protein): an international, multicentre, pragmatic, registry-based randomised trial. The Lancet. 2023 Feb 18;401(10376):568–76. doi:10.1016/S0140-6736(22)02469-2 PubMed PMID: 36708732.

8.	Stoppe C, Patel JJ, Zarbock A, Lee ZY, Rice TW, Mafrici B, et al. The impact of higher protein dosing on outcomes in critically ill patients with acute kidney injury: a post hoc analysis of the EFFORT protein trial. Crit Care. 2023 Oct 18;27(1):399. doi:10.1186/s13054-023-04663-8 PubMed PMID: 37853490; PubMed Central PMCID: PMC10585921.

9.	Haines RW, Prowle JR, Day A, Bear DE, Heyland DK, Puthucheary Z. Association between urea trajectory and protein dose in critically ill adults: a secondary exploratory analysis of the effort protein trial (RE-EFFORT). Crit Care. 2024 Jan 16;28(1):24. doi:10.1186/s13054-024-04799-1 PubMed PMID: 38229072; PubMed Central PMCID: PMC10792897.

10.	Bels JLM, Thiessen S, Gassel RJJ van, Beishuizen A, Dekker ADB, Fraipont V, et al. Effect of high versus standard protein provision on functional recovery in people with critical illness (PRECISe): an investigator-initiated, double-blinded, multicentre, parallel-group, randomised controlled trial in Belgium and the Netherlands. The Lancet. 2024 Aug 17;404(10453):659–69. doi:10.1016/S0140-6736(24)01304-7 PubMed PMID: 39153816.

11.	Schouteden E, Heuts S, Bels JLM, Thiesen S, van Gassel RJJ, Lee ZY, et al. The impact of high versus standard enteral protein provision on functional recovery following intensive care admission: A pre-planned Bayesian analysis of the PRECISe trial. Clinical Nutrition. 2025 May 1;48:153–60. doi:10.1016/j.clnu.2025.03.022

12.	Summers MJ, Chapple L anne S, Karahalios A, Bellomo R, Chapman MJ, Ferrie S, et al. Augmented Enteral Protein During Critical Illness: The TARGET Protein Randomized Clinical Trial. JAMA. 2025 Jul 22;334(4):319–28. doi:10.1001/jama.2025.9110
